# Patterns of intra- and intertumor phenotypic heterogeneity in lethal prostate cancer

**DOI:** 10.1172/JCI186599

**Published:** 2025-06-10

**Authors:** Martine P. Roudier, Roman Gulati, Erolcan Sayar, Radhika A. Patel, Micah Tratt, Helen M. Richards, Paloma Cejas, Miguel Munoz Gomez, Xintao Qiu, Yingtian Xie, Brian Hanratty, Samir Zaidi, Jimmy L. Zhao, Mohamed Adil, Chitvan Mittal, Yibai Zhao, Ruth Dumpit, Ilsa Coleman, Jin-Yih Low, Thomas Persse, Patricia Galipeau, John K. Lee, Maria Tretiakova, Meagan Chambers, Funda Vakar-Lopez, Lawrence D. True, Marie Perrone, Hung-Ming Lam, Lori A. Kollath, Chien-Kuang Cornelia Ding, Stephanie Harmon, Heather H. Cheng, Evan Y. Yu, Robert B. Montgomery, Jessica E. Hawley, Daniel W. Lin, Eva Corey, Michael T. Schweizer, Manu Setty, Gavin Ha, Charles L. Sawyers, Colm Morrissey, Henry Long, Peter S. Nelson, Michael C. Haffner

**Affiliations:** 1 Department of Urology, University of Washington, Seattle, Washington, USA.; 2 Division of Public Health Sciences and; 3 Division of Human Biology, Fred Hutchinson Cancer Center, Seattle, Washington, USA.; 4 Center for Functional Cancer Epigenetics, Dana-Farber Cancer Institute, Boston, Massachusetts, USA.; 5 Center of Molecular and Cellular Oncology, Yale Cancer Center, New Haven, Connecticut, USA.; 6 Department of Medical Oncology, Yale University, New Haven, Connecticut, USA.; 7 AstraZeneca Oncology R&D, New York, New York, USA.; 8 Department of Genome Sciences, University of Washington, Seattle, Washington, USA.; 9 Division of Clinical Research, Fred Hutchinson Cancer Center, Seattle, Washington, USA.; 10 Department of Laboratory Medicine and Pathology, University of Washington, Seattle, Washington, USA.; 11 Department of Anatomic Pathology, University of California, San Francisco, San Francisco, California, USA.; 12 Molecular Imaging Branch, National Cancer Institute, NIH, Bethesda, Maryland, USA.; 13 Division of Hematology and Oncology, Department of Medicine, University of Washington, Seattle, Washington, USA.; 14 Basic Sciences Division and; 15 Translational Data Science IRC, Fred Hutchinson Cancer Center, Seattle, Washington, USA.; 16 Human Oncology and Pathogenesis Program and; 17 Howard Hughes Medical Institute, Memorial Sloan Kettering Cancer Center, New York, New York, USA.

**Keywords:** Cell biology, Oncology, Molecular pathology, Prostate cancer, Urology

## Abstract

Metastatic prostate cancer (mPC) is a clinically and molecularly heterogeneous disease. While there is increasing recognition of diverse tumor phenotypes across patients, less is known about the molecular and phenotypic heterogeneity present within an individual. In this study, we aimed to define the patterns, extent, and consequences of inter- and intratumoral heterogeneity in lethal prostate cancer. By combining and integrating in situ tissue-based and sequencing approaches, we analyzed over 630 tumor samples from 52 patients with mPC. Our efforts revealed phenotypic heterogeneity at the patient, metastasis, and cellular levels. We observed that intrapatient intertumoral molecular subtype heterogeneity was common in mPC and showed associations with genomic and clinical features. Additionally, cellular proliferation rates varied within a given patient across molecular subtypes and anatomic sites. Single-cell sequencing studies revealed features of morphologically and molecularly divergent tumor cell populations within a single metastatic site. These data provide a deeper insight into the complex patterns of tumoral heterogeneity in mPC with implications for clinical management and the future development of diagnostic and therapeutic approaches.

## Abstract 2

This study reveals that, on a molecular level, lethal prostate cancer varies both within and between patients, displaying diverse tumor characteristics that have implications for cancer diagnosis and treatment.

## Graphical abstract

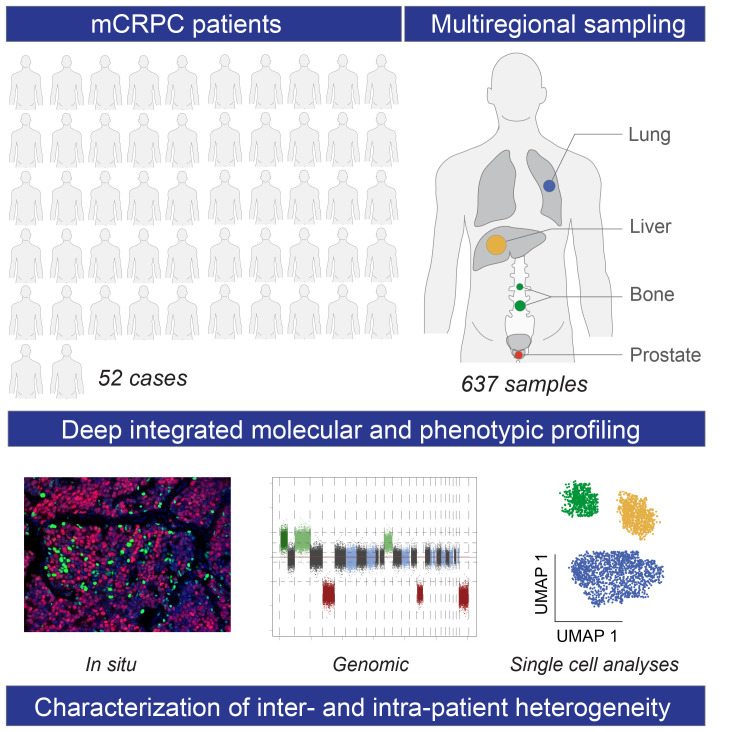

## Introduction

An increasing number of studies spanning diverse cancer types demonstrate that individual cancer cells can show substantial phenotypic and molecular differences across different metastases or even within a given tumor mass (1–6). Such inter- and intratumoral heterogeneity has been discussed as a potential driver of therapy resistance and disease progression (1, 7–9). Broadly, there is a need to formally determine the extent and clinical relevance of tumoral heterogeneity. These characterizations are important since they will guide the development of novel diagnostic tools and help improve therapeutic strategies. However, comprehensively assessing the tumor burden in patients with advanced metastatic disease is challenging due to the difficulties in accessing biospecimen cohorts in which multiple metastatic sites have been sampled from a given patient.

Prostate cancer (PC) is a clinically and molecularly diverse disease and therefore represents a relevant malignancy to study tumoral heterogeneity (10–12). Earlier studies demonstrated that, despite the presence of multifocal genomically distinct tumors in the primary site, the vast majority of distant metastases in a given patient share common genomic alterations, indicating that a single clone in the primary tumor gives rise to all distant metastatic lesions (10, 13–16). Genomic studies have further shown that while key driver gene alterations are shared across different metastases within a patient, there is also evidence of subclonal diversity (10, 13, 15, 17). Clinically, heterogeneous responses to systemic therapies are observed across different metastatic sites, suggesting at least in some patients a higher level of phenotypic diversity and plasticity (18, 19).

Contemporary therapies for metastatic prostate cancer (mPC) exert treatment pressures that result in a diverse spectrum of disease phenotypes characterized by distinct molecular changes and different clinical presentations (20–23). Prior work has shown that loss of prostatic luminal epithelial differentiation and gain of stem-like and neuronal features can be observed in up to 30% of patients with metastatic castration-resistant prostate cancer (mCRPC) (20, 21). Based on these observations, mCRPC can be classified into clinically relevant subtypes by assessing the activity of the androgen receptor (AR) signaling axis and the expression of neuroendocrine (NE) markers (20, 21). This classification enables a contextualization of cell and lineage states: AR+/NE– tumors are characterized by AR expression in the absence of NE differentiation, consistent with a prostatic luminal cell lineage, and comprise the most common molecular phenotype (20, 21). Diverging from this are tumors that gain NE marker expression and/or lose AR or AR activity. AR–/NE– tumors (also termed double-negative PC) show loss of AR expression in the absence of NE marker expression (20, 21). AR+/NE+ tumors, previously termed amphicrine carcinomas, express both AR and NE markers (20, 21, 24–27). Lastly, AR–/NE+ tumors (also termed neuroendocrine prostate cancer [NEPC]) lack AR but show robust NE marker expression and exhibit overlapping features with high-grade NE (small-cell) carcinomas arising in other organ sites (20–22, 28, 29).

Such phenotypic diversity and divergent molecular evolution in response to therapy results in a high level of heterogeneity between cell populations, affecting critical changes in proliferation, metastatic potential, and biological behavior. The resulting inter- and intratumoral heterogeneity potentially poses major challenges for the treatment of patients with mPC. Despite the clinical importance of this phenotypic diversity, few studies have detailed the extent and patterns of inter- and intratumoral heterogeneity in patients with mPC (10, 13, 30, 31).

To address this knowledge gap, we applied multimodal profiling approaches to metastatic tumor tissues that were procured as part of the University of Washington Prostate Cancer Rapid Autopsy Program (16, 20, 21, 32, 33). We integrated in situ analyses and molecular studies across 630 tumor samples from 52 men with lethal mPC to determine the composition and diversity of cellular phenotypes in lethal PC.

## Results

### Patterns of metastatic sites and molecular phenotype distribution.

**Figure 1:**
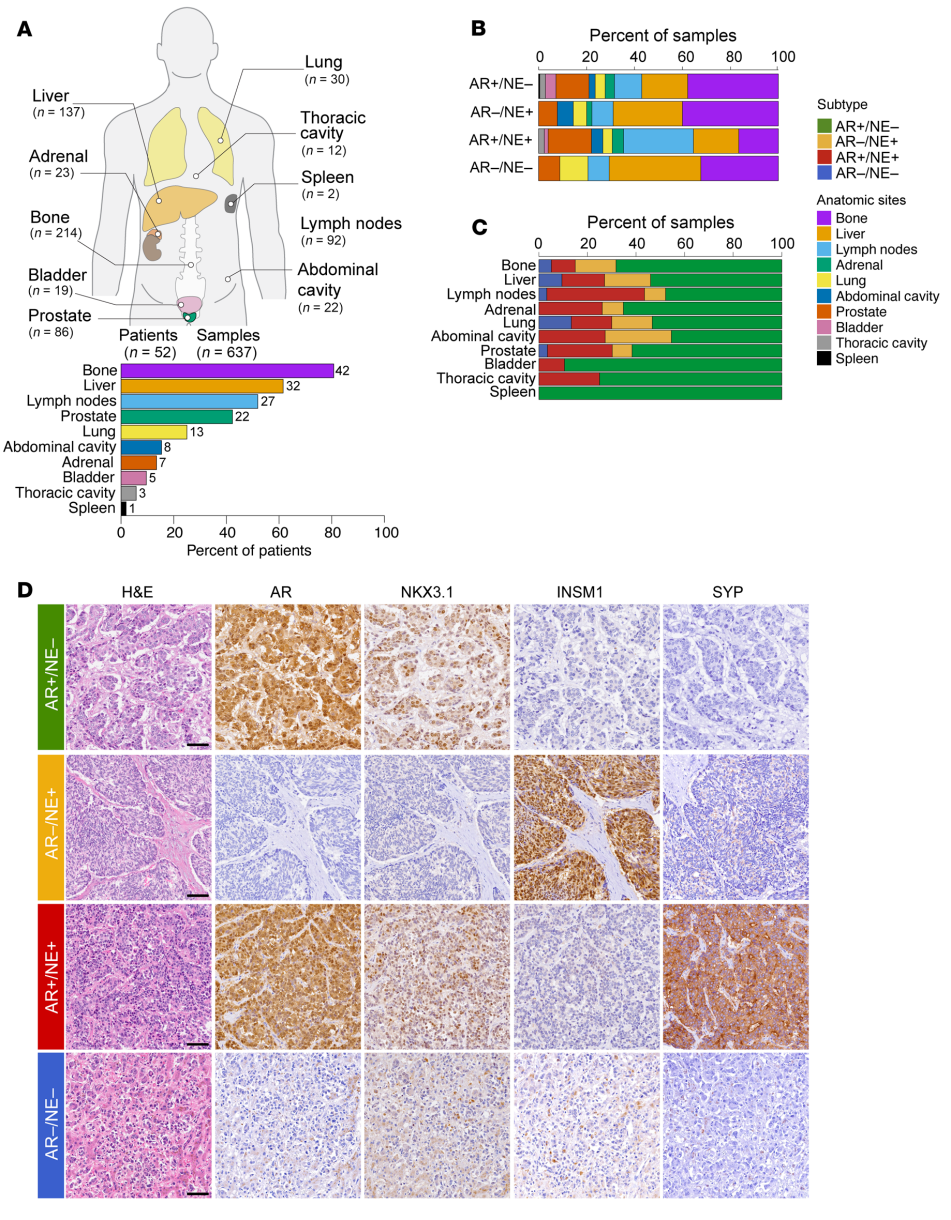
Study cohort and associations between molecular subtype and organ sites. (**A**) Summary of the study cohort. A total of 52 patients and 637 samples were analyzed. Ideogram depicts the distribution of samples across anatomic sites. Bar graph shows the percentage and number of patients with tumor involvement of the listed sites. (**B**) Distribution of organ site involvement across molecular subtypes. (**C**) Proportion of molecular subtypes across organ sites. (**D**) Micrographs of H&E and IHC stains representative of the 4 molecular subgroups. Note that a subset of AR–/NE+ tumors demonstrated robust INSM1 but no SYP expression (as shown here). Scale bars: 100 μm.

To systematically evaluate the diversity of molecular phenotype composition in mPC, we studied 637 PC samples from 52 patients (Figure 1, Supplemental Figure 1, and Supplemental Table 1; supplemental material available online with this article; https://doi.org/10.1172/JCI186599DS1) participating in the University of Washington Prostate Cancer Rapid Autopsy Program from 2003 to 2019 (16, 20). Of these, 51/52 (98%) patients had progressed on prior androgen deprivation or AR pathway inhibitor therapy (Supplemental Table 1). For each patient, a median of 11 tumor samples were included (range 2–37), which represented all metastatic sites, with bone, liver, and lymph nodes being the most frequently involved (Figure 1A). To determine molecular phenotypes across all tissue samples, we constructed tissue microarrays (TMAs) that were stained with a panel of validated antibodies assessing AR signaling (AR, NKX3.1), NE differentiation (insulinoma-associated protein [INSM1], synaptophysin [SYP]), NE-related transcription factors (achaete-scute family BHLH transcription factor 1 [ASCL1], SRY-box transcription factor 2 [SOX2], and forkhead box A2 [FOXA2]), and cell proliferation (Ki-67) (Figure 1 and Supplemental Figure 1). We classified PCs based on AR, NK3 homeobox 1 (NKX3.1), INSM1, and SYP expression into 4 molecular subtypes (33–36). A total of 34 of 52 (65%) patients harbored predominantly AR+/NE– PCs, 12 of 52 (23%) were AR–/NE+, 4 of 52 (8%) were AR+/NE+, and 2 of 52 (4%) classified as AR–/NE–, similar to the distribution of these molecular subtypes observed in other contemporary cohorts (Figure 1 and Supplemental Figure 1) (22, 28, 37, 38). Notably, these molecular subclasses are associated with distinct clinical phenotypes and exhibit differences in the expression of relevant drug targets (Supplemental Figures 2 and 3) (22, 24, 28, 33, 35, 37, 39–44).

Next, we assessed the distribution of different molecular subtypes across anatomic sites (Figure 1, B and C, and Supplemental Figures 1, 4, and 5). We found similar proportions of bone involvement in AR+/NE– (38%) and AR–/NE+ (40%) PCs (Figure 1, B and C). We identified 6 patients with divergent molecular phenotypes, in which at least 1 bone metastasis retained AR expression, while other soft tissue metastases showed loss of AR expression with or without concomitant NE positivity (Supplemental Figure 4). Liver metastases were more common in AR–/NE+ (29%) compared with AR+/NE– (19%) PCs, while the highest rates of liver (38%) and lung (12%) involvement were observed in AR–/NE– PCs (Figure 1, B and C, and Supplemental Table 3). Lymph node metastases were more common in AR+/NE+ (29%) than other subtypes (9%–11%). To formally test the association between molecular subtype and organ site, we applied a mixed-effect regression model. The odds of NE+ disease were significantly higher in liver (OR = 4.8, 95% CI 2.0–11.2, *P* < 0.001), lymph nodes (OR = 3.6, 95% CI 1.5–8.8, *P* = 0.005), and abdominal/retroperitoneal soft tissue (OR = 7.8, 95% CI 2.0–31.3, *P* = 0.004) sites compared with bone.

### Patterns of tumoral heterogeneity and genomic correlates.

**Figure 2:**
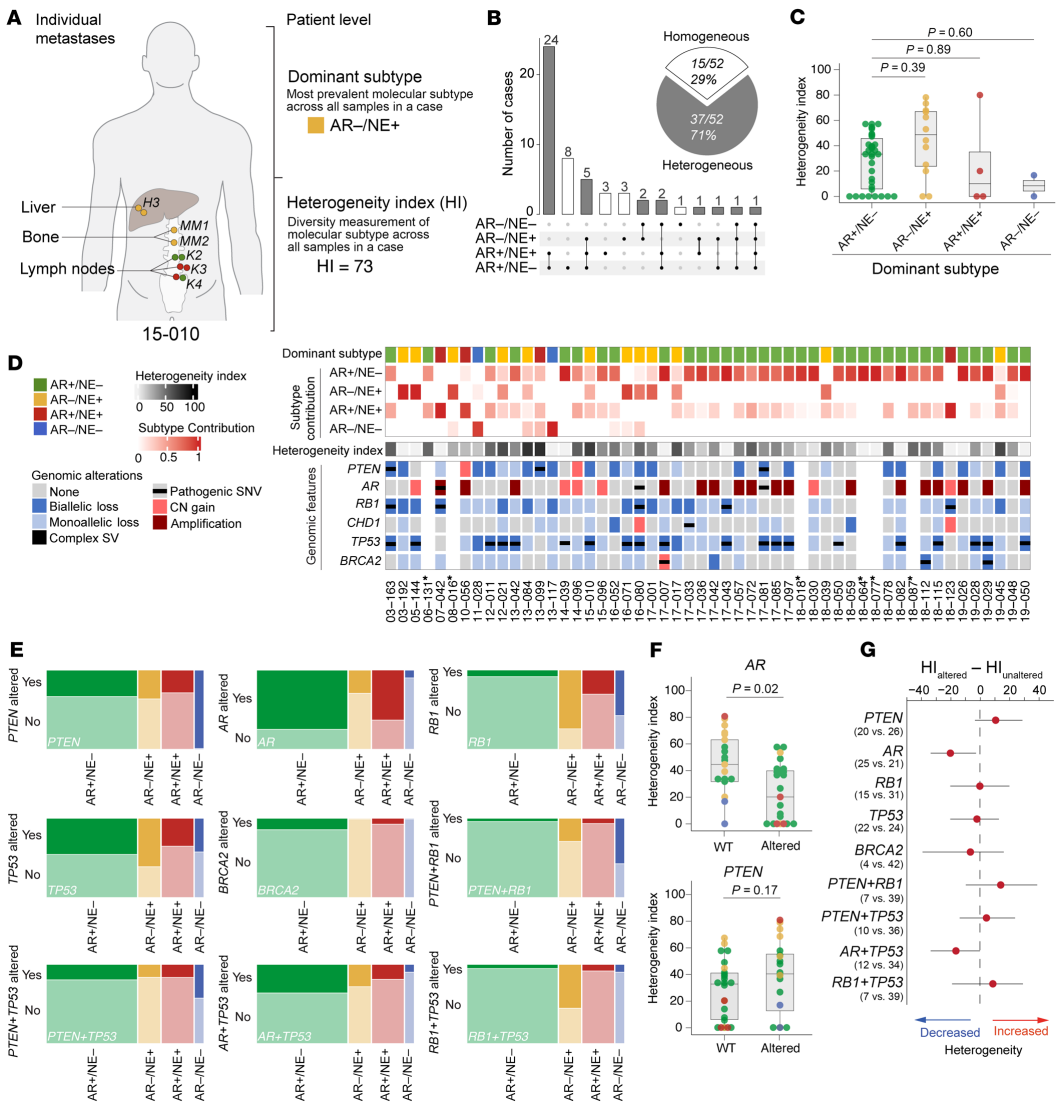
Patterns of subtype heterogeneity and association with genomic alterations. (**A**) Summary of individual sample-level and integrated patient-level assessments of molecular subtypes and subtype heterogeneity, using patient 15-010 as an example. (**B**) Pie chart shows number and percentages of patients with homogenous and heterogeneous molecular subtype distribution. UpSet plot shows the patterns of molecular subtype co-occurrence across all metastatic sites in 52 patients. (**C**) HI distributions across different dominant molecular subtypes. *P* values were derived using the Wilcox-Mann-Whitney test. (**D**) Comutation plot depicting molecular subtype and genomic features of patients included in the study. Each column represents a patient (case IDs are listed at the bottom). Rows show from top to bottom: IHC-derived dominant molecular subtype (defined as the most commonly observed subtype across all metastases); IHC-derived subtype contribution for each of the four subtypes (AR+/NE–, AR–/NE–, AR+/NE+, and AR–/NE–) (heatmap scaled 0–1) shown as the relative fraction of tumor samples from each subtype in a patient; HI (shown as a heatmap scaled 0–100); whole-exome sequencing–derived key genomic alterations (*AR*, *PTEN*, *RB1*, *CHD1*, *TP53,* and *BRCA2*) shared across all metastases (see legend to the left for alteration type). Asterisks indicate no genomic data are available. CN, copy number. (**E**) Mosaic plots illustrating the relative distributions of molecular subtypes (along the *x* axis) and the associated relative distributions of genomic alterations (along the *y* axis) (see Supplemental Table 4 for details). Dark colors indicate the presence of a given genomic alteration, and light colors show absence. Plots are scaled to the total number of samples in each molecular subtype. (**F**) HIs for *AR* and *PTEN* altered and WT patients. *P* values were derived using the Wilcox-Mann-Whitney test. (**G**) Estimated median differences and 95% CIs of HIs between patients with (altered) and without (unaltered) indicated gene alterations (HI_altered_ – HI_unaltered_). Numbers in parentheses represent the number of patients, with the first number indicating altered cases.

Our multitumor and multiregional sampling protocol allowed for an assessment of patterns of intrapatient and intertumoral heterogeneity. To this end, we used the molecular subtype data from each sample to determine the aggregate dominant molecular subtype (i.e., the most common subtype across all samples from a given patient) and the heterogeneity index (HI), which describes the extent of intrapatient subtype heterogeneity across all samples from a given patient (Figure 2A and Supplemental Figure 6). We noted that a minority of patients (15/52, 29%) exhibited a homogeneous phenotype profile (Figure 2B). Overall, 37 of 52 (71%) patients showed an admixture of 2 or more molecular subtypes across different metastatic sites with AR+/NE– and AR+/NE+ PCs co-occurring most frequently (24/37, 65%) followed by patients with AR+/NE–, AR+/NE+, and AR–/NE+ tumors (5/37, 14%) (Figure 2B). The HI showed a broad range between 0% and 80% across patients and was not associated with the number of tumors assessed (Supplemental Figure 6). The median HI was highest in patients with dominant AR–/NE+ PCs, but differences across subtypes did not reach statistical significance (*P* = 0.14) (Figure 2C).

To study the association between key driver genomic alterations, PC subtype, and phenotypic heterogeneity, we determined alterations in *AR*, *BRCA2*, *CHD1*, *PTEN*, *TP53*, and *RB1* in 46 patients (45–50). For all subsequent genomic correlations, only shared alterations were included. This allowed for a global correlation of genomic states and molecular subtype diversity in a given patient. Similar to prior studies, we observed *AR* copy number gains or gain-of-function mutations in 25/46 patients (54%; Figure 2, D and E) (16, 46, 51–53). Co-amplification of the *AR* gene body and a previously identified upstream AR enhancer was detected in 14/18 (78%) evaluable patients (51, 52, 54). Importantly, we observed a high concordance of *AR* genomic alterations across different metastatic sites, with only 3 patients (16-052, 17-033, and 19-048) showing divergent AR changes in single metastases, including low-level copy number gains of ligand binding domain mutations. Biallelic loss of *RB1* was observed in 15/46 (33%) patients; monoallelic *RB1* alterations were present in 22/46 (48%) patients. Biallelic loss or loss-of-function mutations of *TP53* were observed in 22/46 (48%) patients. Across the cohort, 20/46 (43%) patients showed biallelic *PTEN* inactivation (Figure 2, E and F). While *TP53* and *PTEN* alterations were found in AR+/NE– and AR–/NE+ PCs, there was a strong enrichment of *AR* amplifications in AR+/NE–; conversely, *RB1* loss and combined *RB1/TP53* alterations were more prevalent in AR–/NE+ tumors (Figure 2E, Supplemental Figure 6, and Supplemental Table 4). Interestingly, we identified 1 patient (05-144) with uniform AR–/NE+ PC that showed low level *AR* copy number gain and *RB1* and *TP53* loss. Patients with dominant AR+/NE+ tumors had a high rate of *AR* alterations and overall showed a genomic profile similar to patients with dominant AR+/NE– tumors. Patients with dominant AR–/NE– tumors had high rates of *PTEN* and combined *PTEN/RB1* loss (Figure 2, D and E). We next determined the association between subtype HI and genomic features (Figure 2G) and Supplemental Figure 6). We observed that *AR* alterations (*P* = 0.02) were associated with lower HIs, and *PTEN*-altered patients showed marginally and not statistically significantly higher HIs (*P* = 0.17) (Figure 2, F and G). Other genomic alterations did not show robust associations with subtype HI (Figure 2G and Supplemental Figure 6). Collectively, these data suggest that certain genomic contexts are associated with molecular subtypes and intrapatient phenotypic heterogeneity.

### Cell proliferation rates differ across metastases.

**Figure 3:**
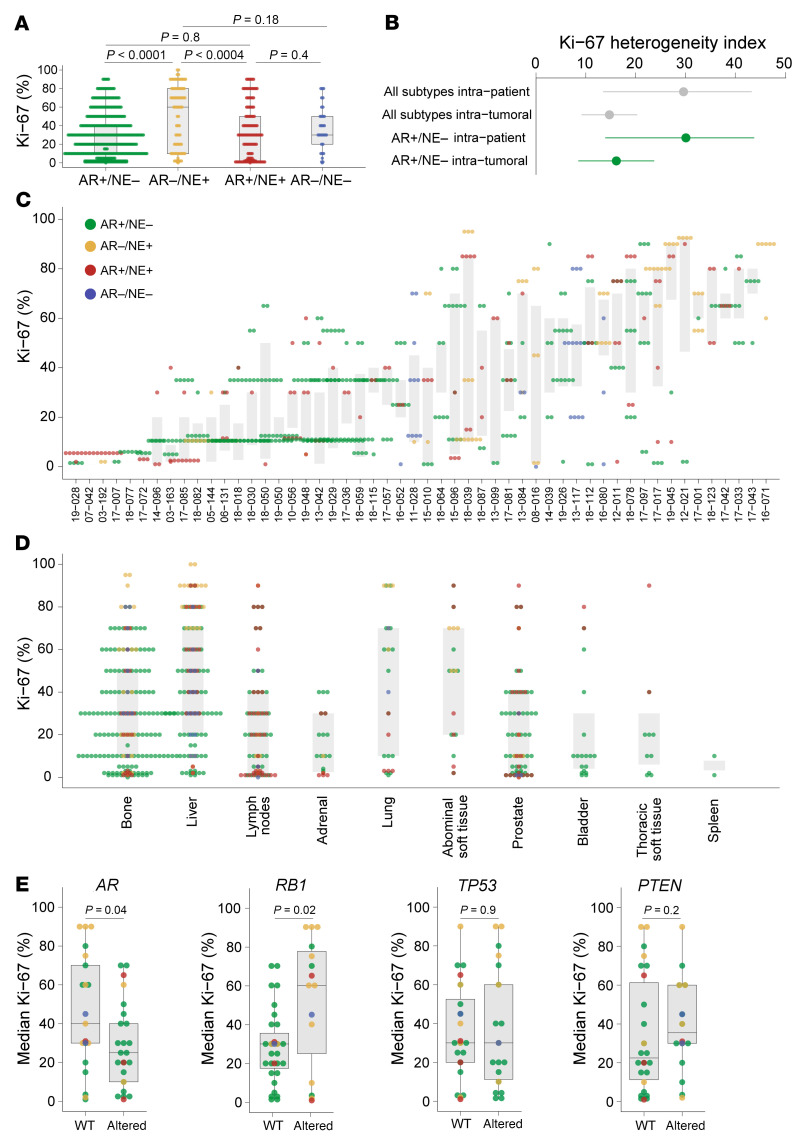
Diversity of cell proliferation patterns across metastatic sites. (**A**) Bar graphs showing the distribution of Ki-67 proliferation indices across different molecular subtypes. *P* values are derived from 2-sample Mann-Whitney rank-sum tests for Ki-67 levels from individual cores. (**B**) Comparison of Ki-67 heterogeneity indices (see Methods) for all patients and AR+/NE– tumors only. (**C**) Scatter plot showing the association between mean Ki-67 indices across all metastases in a given patient. (**D**) Ki-67 indices across different anatomic sites. Each metastasis is shown as a single dot and color coded according to the IHC-determined molecular subtype. (**E**) In box plots, horizontal bars indicate the medians and boxes indicate 25th to 75th percentiles and show Ki-67 indices as a function of genomic status of *AR* and tumor suppressor genes. *P* values were derived using the Wilcox-Mann-Whitney test.

Given the molecular subtype heterogeneity described above, we further sought to investigate differences in proliferative activity among individual metastatic sites. To this end, we assessed Ki-67 proliferation indices, a well-established and clinically used proliferation marker (Figure 3). Across all molecular subtypes, AR–/NE+ PCs exhibited the highest Ki-67 indices (median 60%), followed by AR–/NE– (median 30%), AR+/NE+ (median 30%), and AR+/NE– (median 20%) (Figure 3A and Supplemental Figure 7). Within molecular subtypes, we observed a wide range of Ki-67 positivity (Figure 3 and Supplemental Figure 7). We found that proliferation indices varied substantially across different metastases of the same patient (maximum range of 2%–100%) (Figure 3, B and C, and Supplemental Figure 7). This intertumoral heterogeneity was partly attributable to differences in molecular subtypes, but we also noted marked intertumoral Ki-67 rate differences in metastases of the same molecular subtype (Figure 3, B and C). To quantify Ki-67 diversity both across and within metastases, we applied a separate HI specific to Ki-67 (see Methods) and observed a mean HI of 30 (95% CI 14–43) (Figure 3B). Moreover, there was variability across different anatomic sites, with varying Ki-67 rates observed in lung (median 50%), abdominal soft tissue (median 50%), liver (median 40%), bone (median 30%), thoracic soft tissue (median 20%), lymph nodes (median 20%), and prostate (median 15%) (Figure 3D).

We further explored associations between median Ki-67 levels and genomic alterations and observed lower proliferation indices in PCs with *AR* alterations (median difference = 15%, 95% CI 0%–30%, *P* = 0.04) and higher rates in patients with biallelic *RB1* inactivation (median difference = 30%, 95% CI 5–45, *P* = 0.02) (Figure 3E). While these associations show a trend, they were not considered statistically significant after correction for multiple comparisons. These findings highlight a previously underappreciated intrapatient heterogeneity in proliferation rates across metastatic sites and demonstrate potential trends for associations between cell proliferation, genomic makeup, and molecular heterogeneity.

### Associations between molecular phenotypes, subtype heterogeneity, and clinical features.

**Figure 4:**
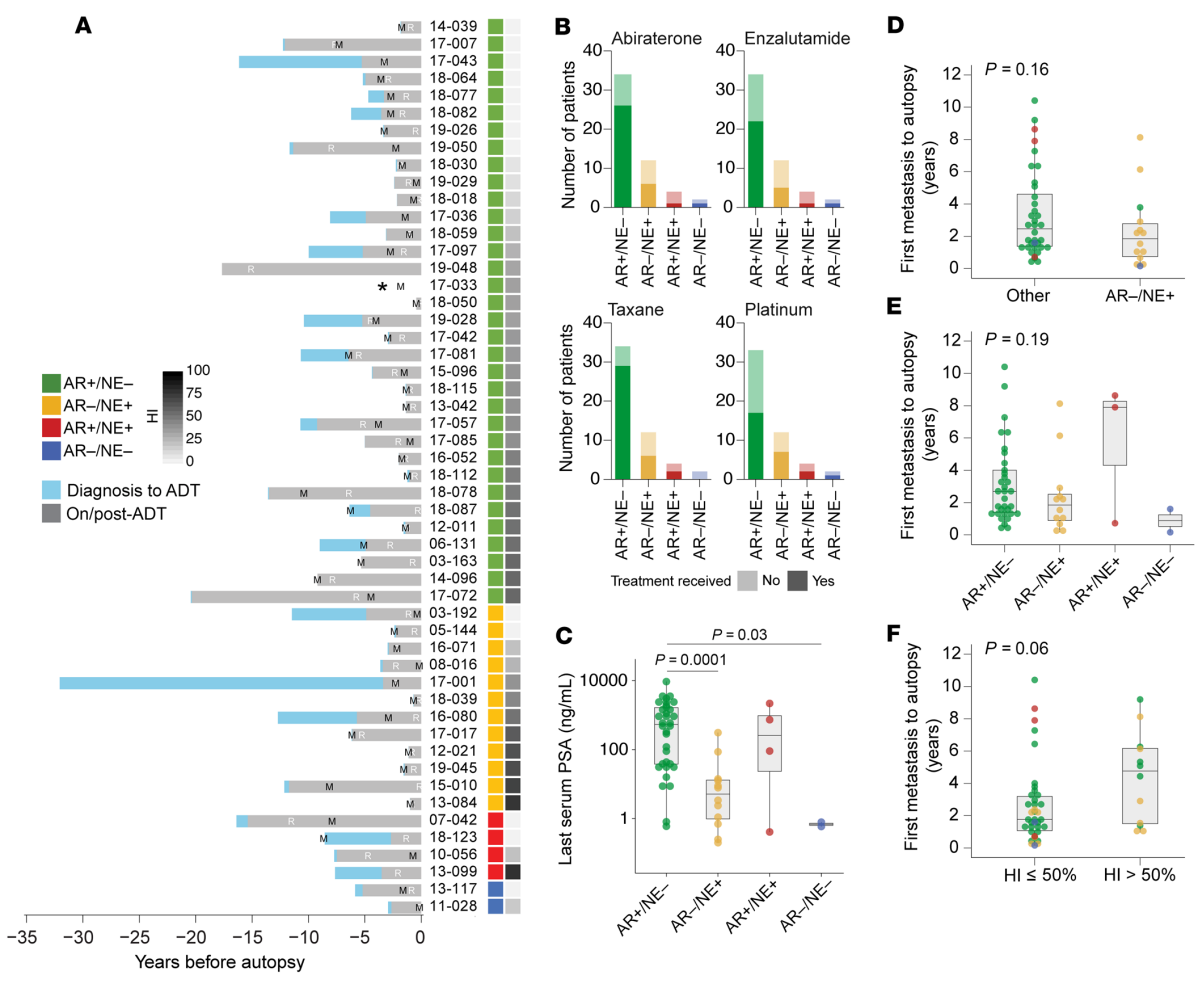
Clinical features associated with molecular subtypes and intrapatient heterogeneity. (**A**) Clinical trajectories for 52 patients included in this study. Bars showing the time from initial diagnosis to death for each patient and are color coded in light blue to indicate the interval from diagnosis to start of androgen deprivation therapy (ADT) and gray for the period after ADT initiation. M indicates the time of first bone metastasis; R indicates first clinical evidence of resistance to ADT. The asterisk indicates that this patient did not receive ADT. Patients are sorted based on dominant molecular subtype (green, AR+/NE–; yellow, AR–/NE+; red, AR+/NE+; and blue, AR–/NE–) and the HI (gray scale heatmap). (**B**) Summary of prior therapies. Stacked bar graphs showing the number of patients that have (dark color) or have not (light color) received the indicated systemic therapies (abiraterone acetate, enzalutamide, and taxane- and platinum-based chemotherapies) as a function of the dominant molecular subtype. (**C**) Last recorded PSA serum levels for each patient broken down by dominant molecular subtype. (**D**) Box plots showing time intervals from first bone metastasis to death for patients with no NE marker positivity compared with patients with any NE marker positivity (INSM1 or SYP H-score ≥ 20). *P* value was derived using the Wilcox-Mann-Whitney test. (**E**) Box plot showing time from bone metastasis to death across all 52 patients stratified by dominant molecular subtype. *P* value was derived using the Kruskal-Wallis test. (**F**) Time interval from time from bone metastasis to death in patients with a HI of greater than 50% or below. Note that corresponding analyses for other time intervals, diagnosis to death, and ADT to death are shown in Supplemental Figure 5D. In box plots, horizontal bars indicate the medians and boxes indicate 25th to 75th percentiles.

The clinical significance of phenotypic heterogeneity in mPC is not well understood. To contextualize our findings, we visualized the diverse clinical histories of men in this cohort (Figure 4A). The intervals between initial PC diagnosis to death (median 5.3 years, IQR 2.2–10.5), first bone metastasis to death (median 2.2 years, IQR 1.2–4.0), and initiation of androgen deprivation therapy to death (median 3.5 years, IQR 2.2–5.5) showed substantial variability (Figure 4A), reflecting the diverse patterns of progression and treatment histories in men with lethal mPC (Figure 4B). Last recorded serum prostate-specific antigen (PSA) values varied significantly but were lowest in AR–/NE+ and AR–/NE– patients (Figure 4C). Further, PSA values were associated with AR expression and Ki-67 positivity but not with HI (Supplemental Figure 8). The presence of an AR–/NE+ tumor showed a numerical but not statistically significant difference in time from bone first metastasis to death (median 2.5 vs. 1.9 years) (Figure 4D and Supplemental Figure 8). In addition, this time interval was numerically shorter in men with AR–/NE+ dominant subtype (median 1.9 years, IQR 0.9–2.5) and AR–/NE– dominant subtype (median 0.9 years, IQR 0.5–1.2) compared with AR+/NE– (median 2.7 years, IQR 1.4–4.0) and AR+/NE+ (median 7.9 years, IQR 4.3–8.3) (Figure 4E). To determine if the level of molecular subtype heterogeneity was associated with outcome differences, we compared patients with high (HI > 50) versus low (HI < 50) molecular heterogeneity at the time of autopsy. Interestingly, we observed a trend toward a shorter duration from first bone metastasis to death in patients with lower heterogeneity (median difference 1.78, 95% CI –0.05–3.88, *P* = 0.06) (Figure 4F).

### Dissecting the molecular heterogeneity of mCRPC at the single-cell level.

**Figure 5:**
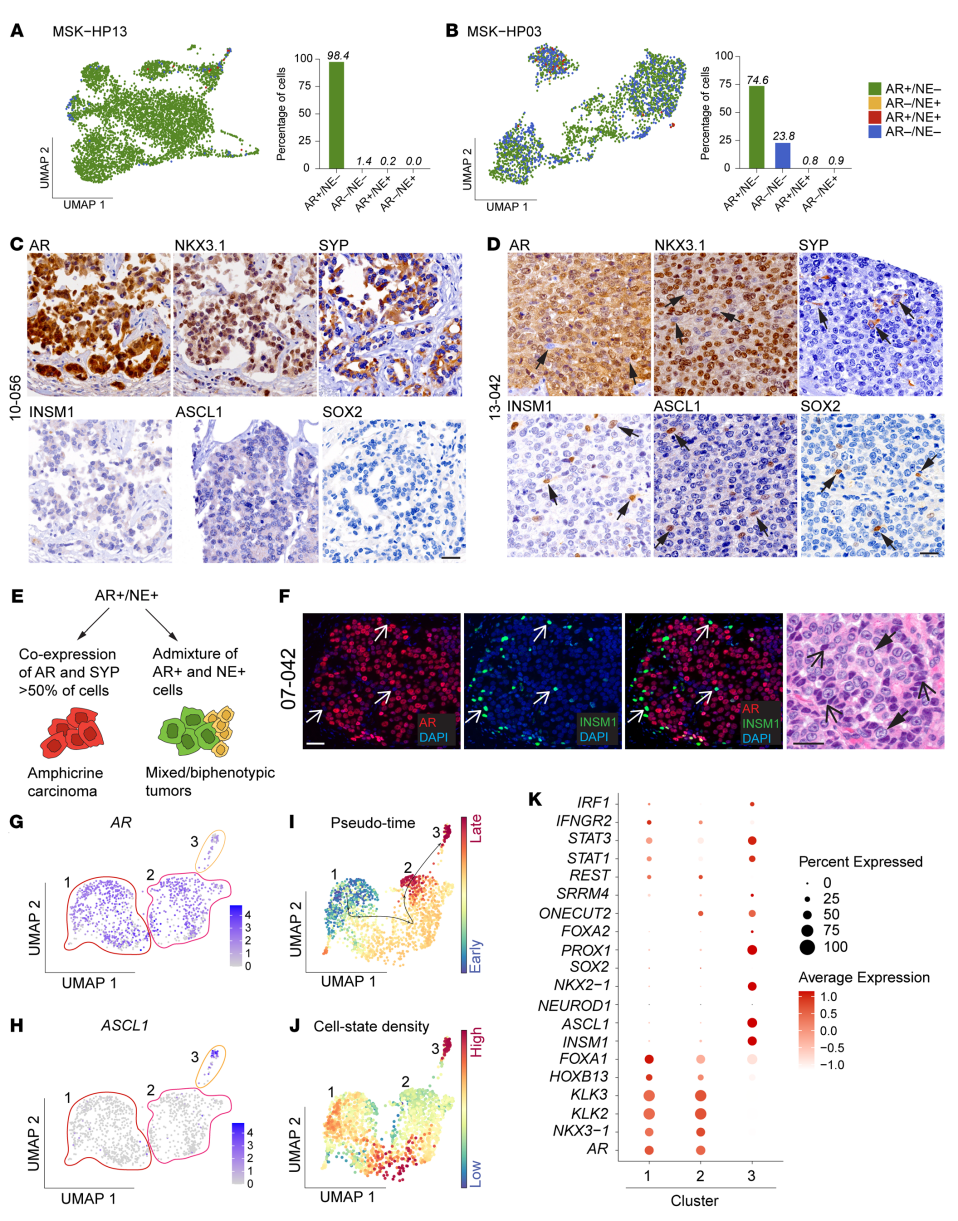
Dissecting molecular subtype pattern at the single-cell level. UMAP and bar graphs depicting molecular subtype composition based on reanalysis of snRNA-seq data of patients with mPC (40). (**A**) Example of a homogeneous AR+/NE– tumor (MSK−HP13). (**B**) A more heterogenous AR+/NE– tumor with admixed AR–/NE– cell populations (MSK−HP03). (**C** and **D**) UMAPs from additional mPCs can be found in Supplemental Figure 8. IHC micrographs of AR (AR, NKX3.1) and NE markers (SYP, INSM1, ASCL1, and SOX2) representative of (**C**) an amphicrine carcinoma (10-056) and (**D**) a mixed/biphenotypic tumor. Note the absence of NE transcription factor expression in the amphicrine carcinoma. Scale bars: 50 mm. (**E**) Schematic showing different cell states in AR+/NE+ tumors. Note that despite widespread positivity for AR and NKX3.1, there are distinct cell populations that are negative for these AR markers but positive for SYP, INSM1, ASCL1, and SOX2 (arrows). (**F**) Coimmunolabeling of mixed/biphasic tumor (07-042) highlights distinct AR–/INSM1+ (arrows) and AR+/INSM1+ (arrowheads) cell populations that show adenocarcinoma and small-cell carcinoma morphology, respectively. Scale bars: 50 μm. (**G** and **H**) Integrated snRNA-seq and snATAC-seq UMAPs showing cell clusters with differential expression of AR and NE markers. Note low-level AR expression but high NE marker expression in cluster 3. (**I**) Pseudo–time analysis using Palantir and (**J**) cell state densities assessment using Mellon showing cell differentiation trajectories across the 3 clusters. (**K**) Bubble plot highlighting differential single-gene expression across the 3 clusters.

To further investigate molecular subtype heterogeneity at the single-cell level, we analyzed previously published single-cell RNA sequencing data and assessed AR and NE positivity using established multigene signatures (40, 49). In predominantly AR+/NE– PCs (*n* = 7), we observed varying degrees of subtype admixture, with AR–/NE– cells being the most commonly detected phenotype (mean 10%, range 0.4%–34.2%) (see Figure 5, A and B, for 2 exemplar cases; Uniform Manifold Approximation and Projections [UMAPs] of all other cases can be found in Supplemental Figure 9). Immunohistochemical analyses further corroborated this finding: Of 347 AR+/NE– PC samples, AR– tumor cells were present in more than 33% (Supplemental Figure 9). Additionally, in predominantly AR–/NE+ (*n* = 3) and AR–/NE– (*n* = 3) tumors, we detected a variable but generally low frequency of subtype heterogeneity at the single-cell level (Supplemental Figure 9).

Across all patients, we observed a significant number of tumors that were positive for both AR and NE markers (AR+/NE+) (Figures 1 and 2). However, we found that AR+/NE+ tumors were a heterogeneous group (Figure 5, C–E). A subset of tumors characterized by the coexpression of AR and NE markers (particularly SYP) was previously termed amphicrine carcinoma to reflect their luminal secretory and NE phenotype (20, 24–27, 55). We identified 3 patients (e.g., 10-056, Figure 5C) that exhibited robust and uniform coexpression of both AR and SYP in over 70% of the same cells, consistent with amphicrine carcinoma (20, 24, 25). Notably, such amphicrine tumors mostly lacked expression of transcription factors associated with NE lineage (e.g., INSM1, ASCL1, and SOX2) but were positive for downstream NE markers such as SYP (Figure 5C). An additional 31 patients showed a different pattern of NE marker expression; instead of homogeneous labeling and coexpression of AR and SYP, we observed reactivity for AR and NE markers in distinct cell populations (e.g., 13-042, Figure 5D). In these AR+/NE+ mixed or biphenotypic tumors, the SYP-positive cells were commonly also positive for INSM1 and often coexpressed NE transcription factors such as ASCL1 and SOX2. These findings suggest that AR+/NE+ tumors can be subdivided into 2 groups: tumors with cells that coexpress AR and NE markers (>70% of cells) and mixed or biphenotypic tumors composed of distinct AR+/NE– and AR–/NE+ cell populations (Figure 5E). In the latter group, the frequency of NE marker–positive cells ranged from 1% to 30%. To formally investigate the coexpression of NE markers and AR in these tumors, we conducted dual-label immunofluorescence microscopy. We observed focal coexpression of AR and INSM1 in 10 out of 31 patients, with varying frequencies of colabeling (mean 3.9%, range 0.5%–50%; Supplemental Figure 10).

Among the AR+/NE+ patients, 07-042 was of particular interest; we identified a distinct AR– but INSM1+ cell population intermixed with predominantly AR and SYP coexpressing cells (Figure 5, F–K, and Supplemental Figure 11). Notably, these AR–/NE+ tumor cells demonstrated morphological features reminiscent of small-cell carcinoma, including high nuclear-to-cytoplasmic ratios, hyperchromatic nuclei, and nuclear molding, whereas the AR+ cells were cytologically consistent with a prostatic adenocarcinoma with open chromatin and prominent nucleoli (Figure 5F and Supplemental Figure 11). To better define the underlying molecular changes and the cellular composition of this biphenotypic tumor, we performed single-nucleus RNA sequencing (snRNA-seq) and single-nucleus assay for transposase-accessible chromatin sequencing (snATAC-seq). Using UMAPs, we identified 3 tumor cell clusters that differed in their expression of AR signaling and NE transcription factors (Figure 5, G and H, and Supplemental Figure 11). Clusters 1 and 2 showed coexpression of *AR* and *SYP* consistent with an amphicrine carcinoma and were negative for ASCL1 and INSM1. Conversely, cluster 3, which was composed of a smaller number of cells corresponding to the INSM1+ cells detected by multiplex immunofluorescence studies (Figure 5F), showed greatly reduced/loss of *AR* but gained in *ASCL1*, *INSM1*, and *PROX1* expression (Figure 5, G–K, and Supplemental Figure 11). Pseudo–time modeling using Palantir demonstrated a cell differentiation trajectory from clusters 1 to 3 (Figure 5I) with the highest uniform cell state densities observed in cluster 3 (Figure 5J) (56). While clusters 1 and 2 showed a high level of concordant gene expression, an abrupt shift in the expression of NE-associated genes (such as *REST*, *FOXA2*, and *SRRM4*) was noted compared with cluster 3 (Figure 5, G–K). Interestingly, we observed a more gradually increased expression from cluster 1 to cluster 3 of *ONECUT2*, a transcriptional regulator previously implicated in NEPC, and a concomitant decrease in the pioneer factor *FOXA1* (57–60) (Figure 5K). Gene set enrichment analyses comparing cells in clusters 2 and 3 showed downregulation of AR response genes in cluster 3 and an increase in genes involved in inflammatory response and interferon signaling, consistent with recent studies implicating JAK/STAT signaling in early lineage transition toward NEPC (49, 61) (Supplemental Figure 11). These transcriptional changes were associated with chromatin alterations assessed by snATAC-seq (Supplemental Figure 11). We further determined the genomic relationship across the 3 clusters by analyzing snATAC-seq data to infer copy number variations (CNVs) (Supplemental Figure 11) and observed shared CNVs across all clusters but also additional CNVs that were only present in clusters 2 and 3. These integrative analyses document the dynamic phenotypic, epigenetic, and genomic changes operative in mPC.

### Tracing molecular and morphologic heterogeneity between and within metastases.

**Figure 6:**
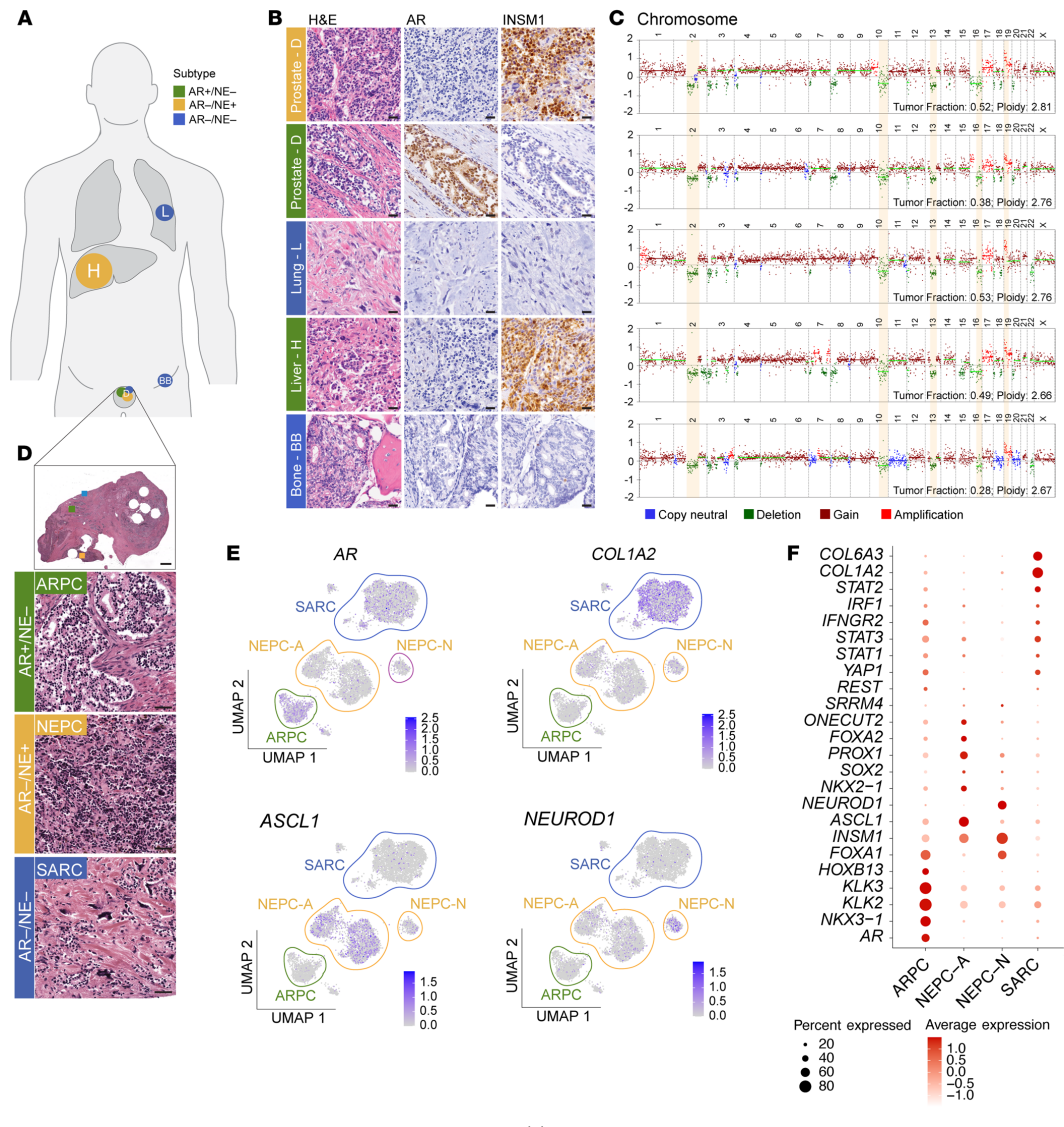
Inter- and intratumoral heterogeneity at the single-patient level. (**A**) Schematic of analyzed metastatic sites and phenotype distribution in patient 13-084. (**B**) Representative micrographs of 5 lesions demonstrating the spectrum of histomorphological and molecular heterogeneity across different tumor sites. (**C**) Somatic copy number profiles derived from WGS demonstrating overlapping copy number changes and limited genomic diversity across phenotypically diverse metastases. Shared copy number changes in key genomic regions are highlighted in yellow. (**D**) Histomorphologic assessment of a prostatic/periprostatic tumor mass shows adjacent AR+/NE– adenocarcinoma (ARPC), AR–/NE+ small-cell carcinoma (NEPC), and AR–/NE– sarcomatoid carcinoma (SARC). (**E**) UMAP based on snATAC-seq data demonstrating 4 tumor cell clusters based on chromatin accessibility pattern and highlighting 2 distinct NEPC clusters that are characterized by ASCL1 (NEPC-A) and NEUROD1 (NEPC-N) expression (both AR–/NE+). (**F**) Bubble plots showing cluster-specific gene expression pattern based on snRNA-seq. Scale bars: 50 μm.

To further illustrate the pronounced phenotypic and molecular heterogeneity observed at the individual patient level, we performed additional integrative molecular studies of phenotypically illustrative cases. For instance, in patient 13-084, histomorphological assessment revealed cribriform adenocarcinoma in the prostate and bone, high-grade NE carcinoma in the liver and prostate, and sarcomatoid carcinoma in a periprostatic soft tissue mass and in the lung (Figure 6, A and B). These morphologically distinct metastases displayed varying patterns of molecular phenotypes: The cribriform adenocarcinoma in the prostate was AR+/NE–, whereas the bone lesion was AR–/NE–. The high-grade NE tumors in the liver and prostate were AR–/NE+. The sarcomatoid carcinoma was AR–/NE–. Bulk whole-genome sequencing (WGS) analyses revealed that, despite these morphologic and molecular differences, driver alterations, including *RB1* and *PTEN* loss, were present in all metastatic sites (Figure 6, B and C). Similarly, in another patient with phenotypically diverse metastases (18-039; Supplemental Figure 12), genomic alterations were concordant across all tumors.

To delve deeper into the molecular tumor features at the single-cell level, we performed snRNA-seq and snATAC-seq on a prostate tissue specimen from patient 13-084 containing adjacent and intermixed AR+/NE– prostatic adenocarcinoma (ARPC), AR–/NE+ high-grade NEPC, and AR–/NE– sarcomatoid carcinoma (SARC) cell populations (Figure 6D and Supplemental Figure 13). UMAP analysis of the snATAC-seq data revealed 4 tumor cell clusters with distinct open chromatin patterns in *AR* (ARPC) and *COL1A2* (SARC). Notably, the 2 additional clusters exhibited differential activity at gene loci encoding the NE transcription factors ASCL1 (NEPC-A) and NEUROD1 (NEPC-N), suggesting the presence of 2 NEPC cell populations with distinct chromatin accessibility patterns identified in prior studies (Figure 6E and Supplemental Figure 13) (31). Importantly, copy number analyses on snATAC-seq data revealed shared CNVs across all clusters that overlap with the CNV profile of the patient’s prostate tumor assessed by bulk WGS (Figure 6C and Supplemental Figure 14). Further, principal component analyses of snATAC-seq data showed that the distinct cell populations in this patient tightly clustered with patient-derived xenograft and cell line models representing the respective molecular subtype (Supplemental Figure 13) (31). Matched snRNA-seq analyses showed expression differences affecting core transcription factors and lineage marker genes across the distinct tumor cell clusters and highlighted cell cluster–specific expression patterns (Figure 6F and Supplemental Figure 14). These studies demonstrate the complexity of coevolving lineage divergent (prostatic luminal epithelial, NE, and mesenchymal/sarcomatoid) cell populations that share a common clonal origin.

## Discussion

There is increasing evidence that treatment pressures exerted by targeted therapies can result in the emergence of cancers with aberrant differentiation and lineage plasticity (29, 62–64). In response to AR-directed therapies, advanced PCs undergo changes in cellular phenotypes, resulting in increased heterogeneity (20, 21, 28, 49, 62). In this study, we aimed to define the patterns, extent, and consequences of tumoral heterogeneity in lethal PC. Combining and integrating in situ tissue-based assessments of protein expression and single-cell sequencing methodologies enabled us to investigate cell populations at both the histomorphologic and molecular levels.

Prior studies have established the evolution and coexistence of tumor cells of different molecular subtypes in mCRPC (20, 22, 28, 31, 49, 65–67). Here, we applied an integrative molecular pathology approach to samples collected as part of a rapid autopsy program. These efforts revealed phenotypic heterogeneity at the patient, metastasis, and cellular levels, with important implications for clinical management and the future development of diagnostics and therapeutics.

Given the molecular diversity observed in this study, our data suggest that in at least a subset of mCRPC patients, a biopsy from a single metastatic site may not adequately represent the phenotypic heterogeneity of a patient’s tumor burden. Previous reports have indicated that genomic driver alterations are largely conserved across different metastatic sites (10, 14–17). Our study supports this conclusion but demonstrates that tumors exhibit a higher level of inter- and intratumoral phenotype variability. This is reflected in the diversity of molecular subtypes and variability of cell proliferation indices. Taken together, these findings support the idea that clinically relevant cellular characteristics can exhibit intrapatient heterogeneity and suggest that evaluating the molecular states of multiple metastatic sites may be needed in certain clinical contexts.

Our assessment of the metastatic tumor burden and molecular phenotyping provides insights into the patterns of metastatic spread among patients with advanced lethal PC. While it was previously suggested that the liver was the predominant site in NEPC cases, we observed significant bone involvement in a substantial number of NEPC patients (68–70). Conversely, in this autopsy cohort, AR+/NE– tumors also showed a high incidence of liver and visceral metastases. These findings indicate a potential shift in metastasis distribution and suggest that the burden of liver metastases might be underestimated in men with advanced mCRPC (68, 69).

Of particular interest are cancers expressing both AR and NE markers (AR+/NE+ subtype). Such tumors can manifest in 2 distinct patterns: Tumors with diffuse coexpression of AR and NE differentiation markers, also known as amphicrine carcinomas, are, despite their NE marker expression, molecularly and clinically similar to AR+/NE– tumors (20, 24–26, 55). Most amphicrine carcinomas show expression of downstream NE markers (such as SYP and chromogranin A) with relatively uniform high expression. NE transcription factors are often negative. This contrasts to mixed/biphenotypic tumors that are composed of intermixed cell populations of AR+/NE– and AR–/NE+ cells. In such patients, the NE cell population often expresses NE transcription factors (e.g., INSM1, ASCL1, and SOX2). Additional studies are needed to investigate the outcome and therapeutic response patterns of amphicrine and mixed/biphenotypic tumors to better define their clinical significance (24, 39).

From a therapeutic perspective, the presence of molecularly diverse tumor cell populations poses a significant challenge. Notably, several drug targets — particularly cell surface proteins used for cancer-specific targeting — are differentially expressed across PC molecular subtypes (Supplemental Figures 2 and 3) (33, 35, 37, 40, 41, 71). Therefore, future treatment strategies must account for the inter- and intratumoral heterogeneity described here. Developing combination therapies that address the unique vulnerabilities and expression patterns of admixed, subtype-divergent cells is essential.

In one patient analyzed in this study, we were able to dissect the molecular features of a mixed/biphenotypic tumor that demonstrated evidence of molecular evolution toward NEPC. While this patient showed molecularly and morphologically distinct cell populations, snRNA-seq and snATAC-seq studies suggested the presence of an evolving NEPC-like cell population characterized by shifts in transcription factor and differentiation marker expression, which was associated with a decrease in *AR* and an increase in *ASCL1*, *PROX1*, and *ONECUT2* expression. Interestingly, during this transition, we observed an increase in cancer cell autonomous expression of gene sets involved in inflammation and interferon signaling. Notably, in cell line and genetically modified mouse models, increased interferon signaling has been linked to lineage plasticity disease states, and preclinical studies indicate that targeting interferon signaling might potentially reverse lineage plasticity (49, 61). This observation suggests that mixed/biphenotypic tumors could represent an intermediate cellular state where therapies aimed at preventing, delaying, or reversing plasticity could be relevant.

Increased levels of genomic tumoral heterogeneity have been linked to higher rates of disease recurrence in localized PC and more broadly associated with aggressive disease behavior in some studies (2, 72–77). However, in other contexts, tumors with extensive genomic subclonal diversity have demonstrated less aggressive disease (3, 4, 72, 78–80). Thus, the current literature indicates a nuanced relationship between tumoral heterogeneity levels and clinical outcomes. In this study, we explored the connection between phenotypic heterogeneity in lethal mPC at the time of death and disease progression from the first metastasis. Unexpectedly, we found that lower HIs at autopsy were associated with a more rapid progression from first metastasis to death. While the relatively small sample size and retrospective nature of the analysis must be considered, our findings suggest that, at least in a subset of patients, aggressive disease may exhibit greater phenotypic homogeneity and only limited inter- and intratumoral heterogeneity. This underscores the importance of the timing of tumor sampling, suggesting that as subclones expand and molecularly distinct cell populations appear, the fittest subclonal population can dominate the tumor burden. Parenthetically, it is also worth noting that certain genomic alterations that are known to be associated with treatment resistance (e.g., *AR* amplification) appear to restrict subtype heterogeneity.

There are several limitations to our study. We focused on analyzing advanced lethal mPC samples from a rapid autopsy cohort at a single institution. While this represents the most extensive assessment of phenotypic heterogeneity in mPC to date, the retrospective nature and the relatively small overall number of patients limit the statistical power for assessing clinical associations. Hence, prospective studies in earlier disease settings are crucial to validate and refine the findings presented here and to understand the impact of tumoral heterogeneity on treatment response. Given the large number of individual metastases included in this study, we were unable to perform detailed genomic analyses on each sample. Instead, we focused on shared genomic features for the correlative analyses presented in Figure 2. Future studies are necessary to explore how individual genomic and epigenetic alterations associate with different molecular subtypes on the single metastasis level to further dissect their contribution to subtype diversity. While our study offers insights into the intricate composition of mPC tumors, our primary focus was on discerning patterns within established tumor cell intrinsic cell states. Future studies are necessary to delineate the pattern of other molecular subtypes and to evaluate the diversity of therapeutic targets. Furthermore, this study focuses on the assessment of tumor cell intrinsic features, and future studies need to address the diversity of tumor microenvironments and their contribution to cellular plasticity in mPC.

## Methods

### Sex as a biological variable.

Since PC affects only males, our study cohort consisted exclusively of male patients.

### Tissue specimens.

Tissue samples were collected from men who succumbed to mPC and who participated in the University of Washington Prostate Cancer Rapid Autopsy Program (16, 21, 30). Procedures were carried out with a mean postmortem interval of 4.9 hours (range 2–23 hours). Following a standard protocol, all grossly identifiable soft tissue and visceral metastases were sampled. Additionally, systematic biopsies were obtained to sample the vertebral and nonvertebral bones. These extensive sampling efforts resulted in a mean number of 120 tissue samples per patient across up to 18 anatomically distinct sites. All bone specimens underwent decalcification with 10% formic acid before paraffin embedding.

### Histopathological review and TMA construction.

H&E-stained FFPE tissue sections from 52 patients (see Supplemental Table 1 for demographic information) were evaluated. Based on the presence of tumor and morphological features, TMAs with a core diameter of 1 mm were constructed as described previously (33, 36, 71). Each metastatic site was sampled with at least 2 cores. For blocks that showed more than 1 distinct morphology, multiple regions of the tumor were sampled.

### Immunohistochemical staining and evaluation.

For all immunostaining experiments, 5 μm thin adjacent sections of TMAs were stained for AR, NKX3.1, SYP, INSM1, ASCL1, SOX2, FOXA2, and Ki-67 using previously validated antibodies (antibody specifications are listed in Supplemental Table 2). In brief, FFPE sections were dewaxed and rehydrated following standard protocols. Antigen retrieval consisted of either steaming for 45 minutes in Target Retrieval Solution (S1700; Agilent) or steaming for 30 minutes in citrate buffer (H-3300-250; Vector Laboratories). Slides were then washed and equilibrated in TBS-Tween buffer (Sigma) for 10 minutes. Primary antibodies were applied, and immunocomplexes were visualized using the secondary antibodies as specified in Supplemental Table 2. All tissue sections were counterstained with hematoxylin, and slides were digitized on a Ventana DP 200 Slide Scanner (Roche). TMAs were reviewed, and immunoreactivities were scored in a blinded manner using a previously established H-score system, whereby the optical density level (“0” for no brown color, “1” for faint and fine brown chromogen deposition, and “2” for prominent chromogen deposition) was multiplied by the percentage of cells at each staining level, resulting in a total H-score (range 0–200) for each core (33, 36, 71). For Ki-67, any nuclear positivity was counted, and the percentages of positive nuclei are shown (range 0%–100%). For subtype classification, AR+ tumors were defined by expression of AR and/or NKX3.1 with an H-score of greater than or equal to 20; NE+ tumors were defined by a SYP and/or INSM1 H-score of greater than or equal to 20 as described previously (36, 45, 71). Note that IHC data for 16 patients were presented in a prior manuscript from our groups (40).

### Immunofluorescence staining.

Multiplexed immunofluorescence colabeling experiments with AR-specific (Cell Signaling Technologies; 5153T) and INSM1-specific (Santa Cruz; sc-271408) antibodies were carried out on archival 5 μM FFPE tissues. The staining protocol consisted of 2 sequential staining steps, each with tyramide-based signal amplification using the Tyramide SuperBoost kit (Thermo Fisher) as described previously (33). Dewaxed slides were first subjected to steaming for 45 minutes in Target Retrieval Solution (S1700; Agilent) and incubated with AR-specific antibodies (1:100). Signal amplification was carried out by first incubating slides with PowerVision Poly-HRP Anti-Rabbit (Leica) secondary antibodies followed by Tyramide568 (Tyramide SuperBoost kit; Thermo Fisher) according to the manufacturer’s protocols. Slides were then stripped by steaming in citrate buffer (Vector) for 15 minutes and subsequently incubated with INSM1-specific antibodies (1:50) followed by PowerVision Poly-HRP anti-mouse (Leica) secondary antibodies and Tyramide488 (Tyramide SuperBoost kit). Tissues were counterstained with DAPI, mounted with Prolong (Thermo Fisher), and imaged on a Nikon Eclipse E800 microscope equipped with a Zeiss AxioCam HRm, 14-bit monochrome camera. All image analyses were carried out using QuPath (v0.3.0) (81).

### Genomic analyses.

Areas of interest were identified on H&E slides and macrodissected from adjacent slides as described previously (13). For multiregional sequencing studies, genomic DNA was extracted using the GeneRead FFPE kit (Qiagen) following the manufacturer’s instructions. DNAs were subjected to paired-end (2 × 150 bp) sequencing on an Illumina HiSeq instrument to an average coverage of 3.05×. For all other genomic analyses, previously published whole-exome sequencing data were used (16, 21, 35). Genomic alterations, which were shared by all metastases in a given patient, were analyzed. Single nucleotide variant (SNV) calling was performed using MuTect 2 (GATK version 4.1.8.1), Strelka 2 (version 2.9.2), and VarScan 2 (version 2.4.4) (82–84). Insertions and deletions (Indel) were called using SvABA (85). All SNV and Indel calls were annotated using ANNOVAR (release 20200607) (86). TitanCNA version 1.23.1 was used for copy number calling (87). Gene-level copy number calls were derived from TitanCNA’s segments using GenomicRanges version 1.38.0. Gene-level copy number calls were converted to ploidy-adjusted copy number (PACN) using TitanCNA’s estimated sample ploidy. The following thresholds were used to define copy number events; amplification: PACN ≥ 2.5, gain: PACN ≥ 1.5, deletion: PACN ≤ 0.5, and homozygous deletion: PACN = 0.

### Bulk and single-nuclei RNA-seq and ATAC-seq.

Bulk RNA-seq data from the Stand Up To Cancer/Prostate Cancer Foundation study was processed as described previously (21, 33, 88). Gene abundance was quantified using GenomicAlignments (89). Molecular subtype classification (AR/NE status) was performed as described previously (20). Differential gene expression between molecular subgroups was assessed using limma (90) and refined to cell surface (91) and tier 1 druggable targets (92). For single-nuclei sequencing, nuclei were extracted from frozen sections (2 × 50 m) of archival autopsy tissues and processed as described previously (31). For snATAC-seq, nuclei were transposed according to the OMNI-ATAC protocol (93). Around 7,000 cells were targeted for each sample and processed according to the 10× Genomics single-cell ATAC-seq sample preparation protocol (Chromium Single Cell ATAC Library & Gel Bead Kit; 10x Genomics). For snRNA-seq, nuclei were prepared the same way and processed in parallel using the 10× Genomics snRNA-seq protocol (Chromium Single Cell 3′ v2 Reagent Kit; 10x Genomics). snRNA-seq data were preprocessed using the Cell Ranger (10x Genomics) to obtain unique molecular identifier (UMI) counts for each gene. Cells with less than 200 genes expressed (UMI > 0) or cells with greater than 80% UMIs from mitochondrial genes were excluded. Filtered data were further normalized and scaled using Seurat (94). In addition to de novo–generated data, a previously published set of single-cell RNA-seq data from CRPC patients was included (40). Principal component analysis was performed using the first 50 principal components, and the UMAP dimension reduction technique was used for visualization. The Seurat function AddModuleScore() was used to generate composite scores for each individual cell with previously established gene sets indicative of NE and AR pathway activity (21, 22, 95). Cells with composite scores greater than 0.1 for either the AR or NE signature were correspondingly classified as AR+/NE+, AR–/NE+, AR+/NE–, and AR–/NE–. Unsupervised clustering was performed using the “FindClusters” function in the Seurat R package with a parameter of resolution of 0.8. Differential expression analyses between clusters were performed with the FindMarkers() function using the Wilcoxon’s rank-sum test. False discovery rate was then calculated to account for multiple testing. Single-cell ATAC-seq data were processed using the Cell Ranger ATAC pipeline v1.1.0. Any cell that had fraction of reads in peaks (FriP) less than 0.2 or total fragments less than 1,000 was removed from the analysis. Detection of CNVs from snATAC-seq data was performed as described previously (31). Cell fate and cell state density analyses were performed on snRNA-seq data using Palantir and Mellon (56, 96).

### Statistics.

Associations between median biomarker H-scores from a given patient and postmortem intervals were evaluated within subtypes using linear regressions. An intrapatient HI for subtype was quantified using hypergeometric probabilities of randomly paired tumor samples that have discordant subtypes. This measure of heterogeneity is highly correlated with other measures (e.g., Shannon index and Simpson index) while accounting for finite sampling of molecular subtypes from the same patient. Association between median HI for subtype and dominant subtype was evaluated using the Kruskal-Wallis test; pairwise differences were evaluated using the Wilcox-Mann-Whitney test adjusted for multiple comparisons using Holm’s method. Association between HI for subtype and genomics features was evaluated using the Wilcox-Mann-Whitney test. We quantified an intrapatient HI for Ki-67 positivity using hypergeometric probabilities to randomly sample pairs of tumors and determine the likelihood of discordant positivity (i.e., 1 with level ≤20% and 1 with level >20%). Association between HI for Ki-67 positivity and subtype was evaluated using the Kruskal-Wallis test; pairwise differences were evaluated using the Wilcox-Mann-Whitney test adjusted for multiple comparisons using Holm’s method. Similarly, an intratumoral HI for Ki-67 positivity was quantified by randomly sampling pairs of tumor samples from the same tumor block. Intrapatient and intratumoral HIs for Ki-67 positivity were summarized across patients using 1,000 bootstrap samples and calculating accelerated 95% CIs using the “bcanon” function in the bootstrap R package (96). Association between maximum Ki-67 levels and shared genomic features was evaluated using the Wilcox-Mann-Whitney test accounting for multiple comparisons using Bonferroni’s method (*P* < 0.05/21 = 0.0024 was considered statistically significant). Association between the last serum PSA level or clinical durations (between selected events and autopsy) and dominant subtype, the presence of any NE metastases, and HI for subtype greater than 50% was evaluated using the Kruskal-Wallis test; pairwise differences were evaluated using the Wilcox-Mann-Whitney test adjusted for multiple comparisons using Holm’s method.

### Study approvals.

The study was approved by the University of Washington (IRB#2341) and Fred Hutchinson Cancer Center (IRB#10706) Institutional Review Boards. All participating men provided written informed consent for a rapid research autopsy and tissue procurement.

### Data availability.

Sequencing data generated as part of this proposal are available in public repositories (GSE292195). All immunohistochemical data on the single-case level are provided in the Supporting Data Values file. Analytic code can be requested from the corresponding author.

## Author contributions

MPR and ES designed research studies, conducted experiments, acquired data, analyzed data, and wrote the manuscript. MPR, ES, and MCH reviewed the H&E-stained FFPE tissue sections and TMAs. RG designed research studies, conducted experiments, analyzed data, and wrote the manuscript. RAP, BH, and IC conducted experiments, acquired data, analyzed data, and wrote the manuscript. M Tratt, HMR, CKCD, SH, and MS analyzed data and wrote the manuscript. PC conducted experiments, acquired data, analyzed data, provided data, and wrote the manuscript. MMG and PG acquired, analyzed, and provided data. XQ and JYL conducted experiments, acquired data, and analyzed data. YX acquired data, analyzed data, and wrote the manuscript. SZ and JLZ conducted experiments, acquired data, analyzed data, provided reagents and data, and wrote the manuscript. MA analyzed data, provided data, and wrote the manuscript. C Mittal acquired and analyzed data. YZ analyzed data. RD and MC conducted experiments and provided reagents. TP analyzed and provided data. JKL acquired data, analyzed data, provided reagents and data, and wrote the manuscript. M Tretiakova provided reagents and wrote the manuscript. FVL conducted experiments, acquired data, and provided data. LDT and MP conducted experiments, acquired data, provided reagents, and wrote the manuscript. HML and LAK conducted experiments, acquired data, provided reagents and data, and wrote the manuscript. HHC, EYY, RBM, JEH, DWL, EC, and MTS acquired data, provided reagents and data, and wrote the manuscript. GH acquired, analyzed, and provided data and wrote the manuscript. CLS designed research studies, acquired data, provided reagents and data, and wrote the manuscript. HL designed research studies, acquired data, analyzed data, provided reagents, and wrote the manuscript. PSN designed research studies, acquired data, analyzed data, provided reagents and data, and wrote the manuscript C Morrissey and MCH designed research studies, conducted experiments, acquired data, analyzed data, provided reagents and data, and wrote the manuscript.

## Supplementary Material

Supplemental data

Supplemental tables 1-4

Supporting data values

## Version 1 06/10/2025

In-Press Preview

## Version 2 08/01/2025

Electronic publication
