# Supplementary material for: Patterns of intra- and intertumor phenotypic heterogeneity in lethal prostate cancer
Source: J Clin Invest. 2025 Jun 10;135(15):e186599. doi: 10.1172/JCI186599 (PMC12321404; doi:10.1172/JCI186599)
Supplement: Supplemental data [file jci-135-186599-s062.pdf]

# SUPPPLEMENTARY DATA

## Patterns of intra- and inter-tumor phenotypic heterogeneity in lethal prostate cancer

Martine P. Roudier, Roman Gulati, Erolcan Sayar, Radhika A. Patel, Micah Tratt, Helen M. Richards, Paloma Cejas, Yingtian Xie, Brian Hanratty, Samir Zaidi, Jimmy L. Zhao, Mohamed Adil, Chitvan Mittal, Yibai Zhao, Ruth Dumpit, Ilsa Coleman, Jin-Yih Low, Thomas Persse, Patricia Galipeau, Gavin Ha, John K. Lee , Maria Tretiakova, Meagan Chambers, Lori Kollath, Funda Vakar-Lopez, Lawrence D. True, Marie Perrone, Hung-Ming Lam, Lori A. Kollath, Chien-Kuang Cornelia Ding, Stephanie Harmon, Heather H. Cheng, Evan Y. Yu, Robert B. Montgomery, Jessica E. Hawley, Daniel W. Lin, , Eva Corey, Michael T. Schweizer, Manu Setty, Charles L. Sawyers, Colm Morrissey, Henry Long, Peter S. Nelson, Michael C. Haffner

**Supplementary Figure 1**. *Roudier et al.*

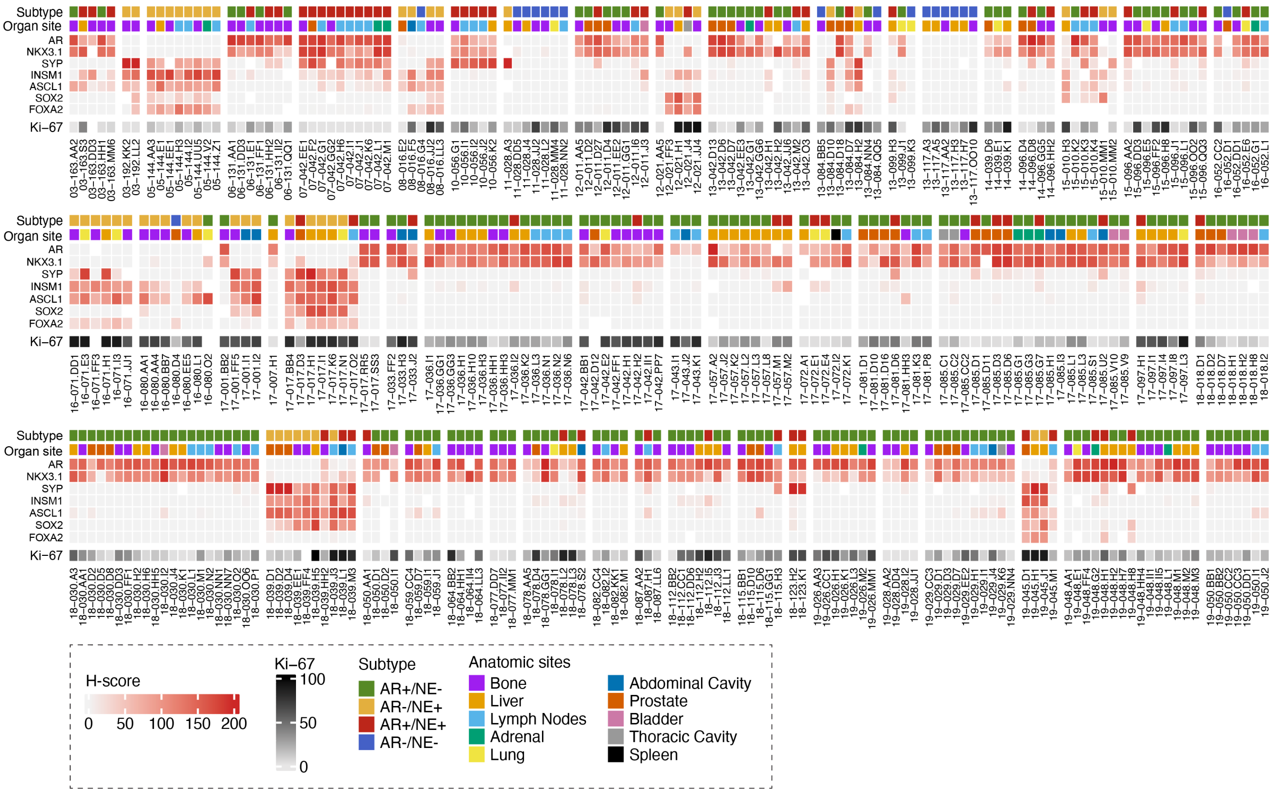


**Supplementary figure 1.** Landscape of inter- and intra-patient subtype diversity in mPC. Heatmap shows protein expression of AR-signaling (AR, NKX3.1), neuroendocrine markers (SYP, INSM1, ASCL1, SOX2, FOXA2) based on semiquantitative H-scoring (range 0-200, gray to red) and Ki-67 percent positive cells (range 0-100, gray to black). Tumors from the same patients are clusters together. White boxes indicate sites with missing data. Anatomic sites, molecular subclass and histomorphology are color coded according to the legend. Note that 16 patients from this cohort have also been analyzed in a prior study (40).

**Supplementary Figure 2**. *Roudier et al.*

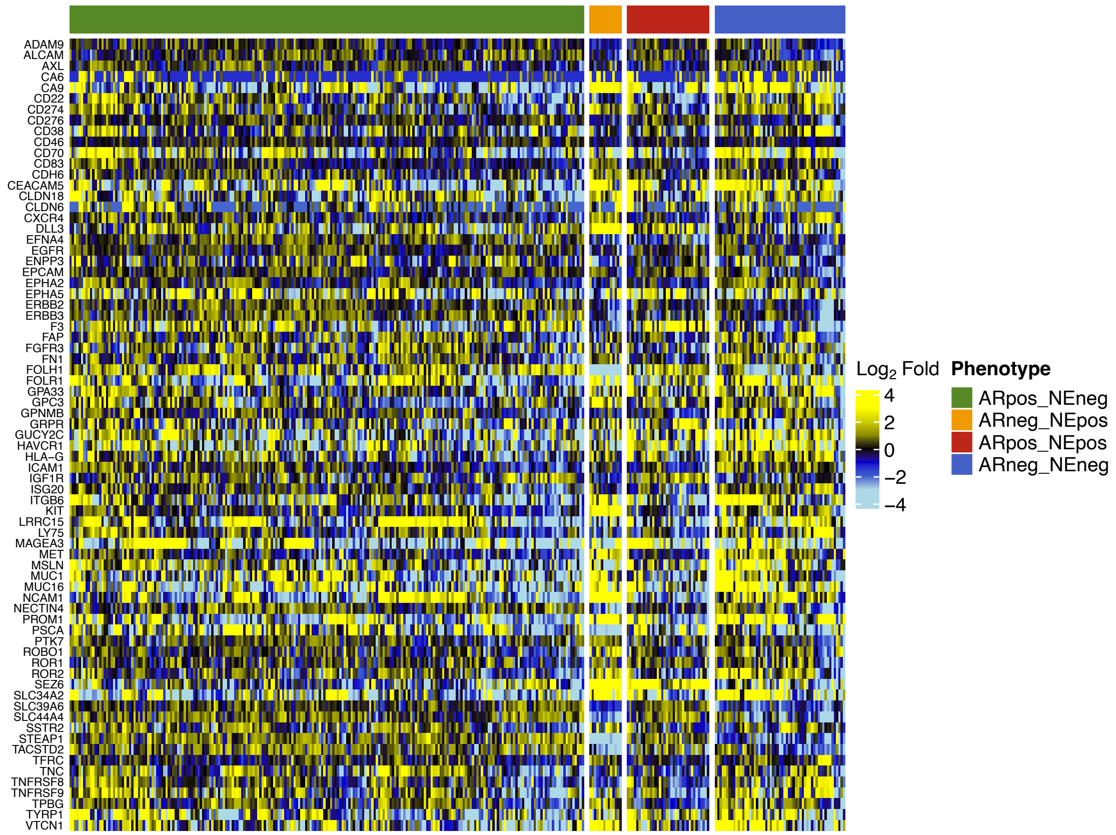


**Supplementary Figure 2.** Heatmap shows expression of clinical cell surface targets across molecular subtypes based on re-analysis of the previously published SU2C International Dream Team RNA-seq dataset (*Abida et al., PNAS, 2019*).

**Supplementary Figure 3**. *Roudier et al.*

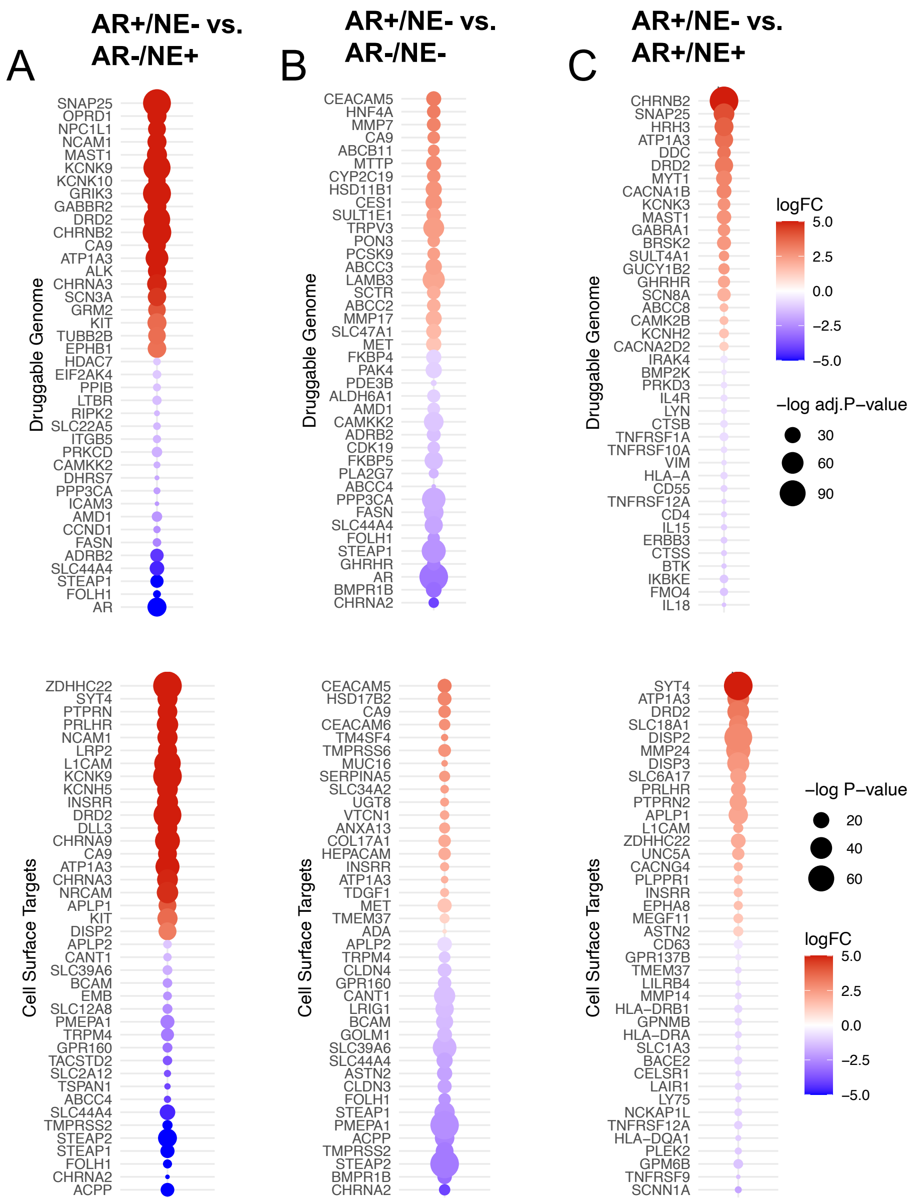


**Supplementary Figure 3.** Heatmap of top 20 differentially expressed genes with annotated drug target properties from the druggable genome database (*top*) and cell surface targets (*bottom*) between **A.** AR+/NE- vs. AR-/NE+; **B.** AR+/NE- vs. AR-/NE-; **C.** A. AR+/NE- vs. AR+/NE+ based on re-analysis of the previously published SU2C International Dream Team RNA-seq dataset (*Abida et al*., *PNAS, 2019*).

**Supplementary Figure 4**. *Roudier et al.*

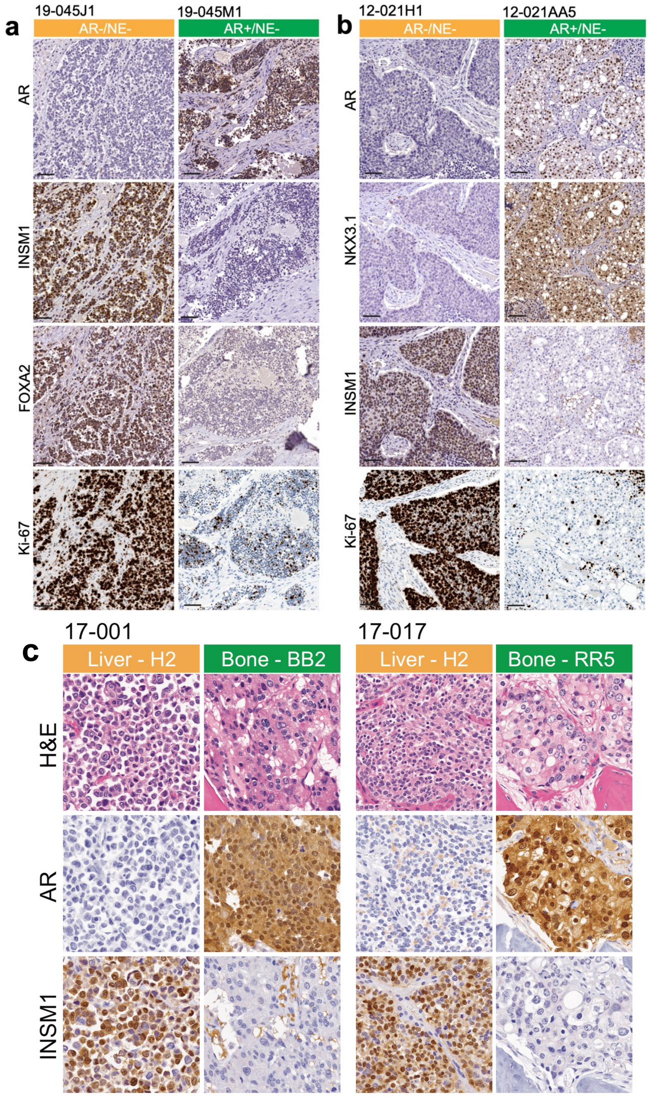


**Supplementary figure 4. A., B.** Representative micrographs of two rapid autopsy cases with intra-tumoral heterogeneity. Note that both cases 19-045 and 12-021 show both adenocarcinoma (AR+/NE) and high-grade neuroendocrine carcinoma (AR-/NE+) in different metastatic sites. **C.** Representative micrographs of hematoxylin and eosin (H&E) and AR and INSM1 IHC in rapid two autopsy cases with divergent differentiation in bone and liver metastases. Note that, liver metastases showed molecular and morphological features of AR-/NE+ high-grade neuroendocrine carcinoma, whereas bone metastases were AR+/NE- and showed adenocarcinoma morphology. Scale bars denote 50 $\mu$m.

**Supplementary Figure 5**. *Roudier et al.*

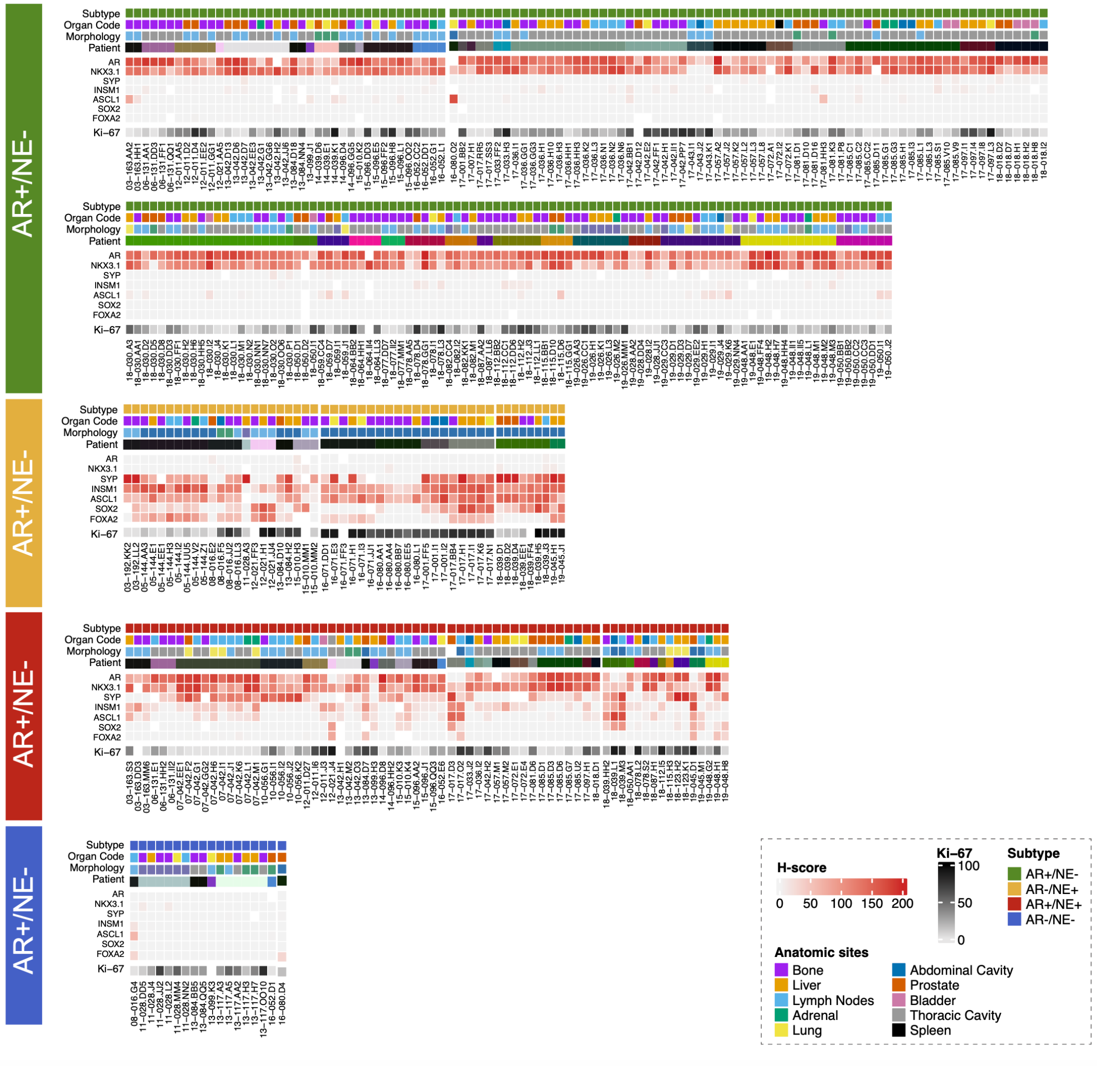


**Supplementary figure 5.** Heatmap (based on *Figure 1C*) of immunohistochemical and phenotype data clustered based on molecular phenotype.

## **Supplementary Figure 6**. *Roudier et al.*

**a**

$$
\begin{aligned}
\mathrm{HI} &= \Pr(\text{samples are different subtypes}) \\
&= 1 - \Pr(\text{samples are the same subtype}) \\
&= 1 - \big[\Pr(\text{samples are both AR−NE+}) \\
&\qquad + \Pr(\text{samples are both AR+NE−}) \\
&\qquad + \Pr(\text{samples are both AR+NE+})\big] \\
&= 1 - \left[\left(\frac{4}{10}\times\frac{3}{9}\right) + \left(\frac{3}{10}\times\frac{2}{9}\right) + \left(\frac{3}{10}\times\frac{2}{9}\right)\right] \\
&= \quad 0.73 * 100 = 73
\end{aligned}
$$

**b**

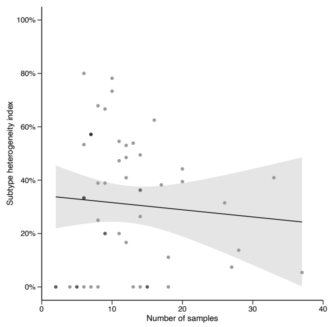


**c**

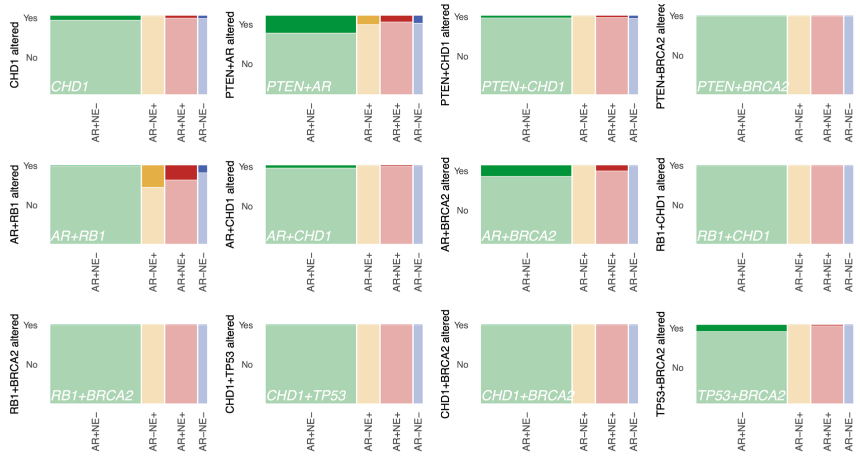


**d**

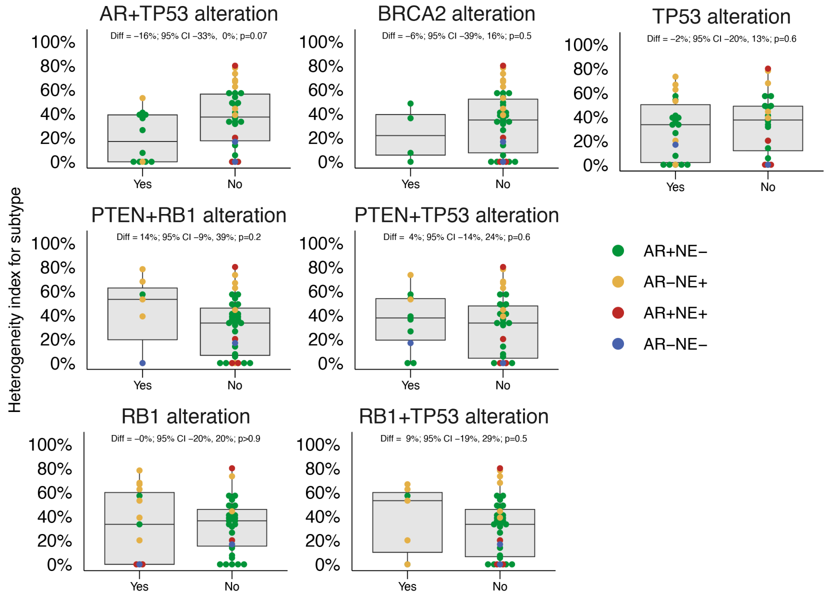


**Supplementary figure 6. A.** Formula for the calculation of the heterogeneity index (HI) using data from case 15-010 as an example (*see Figure 2*). **B.** Scatter plot shows lack of correlation between number of metastasis and the subtype HI. **C.** Mosaic plots depict relative distributions of molecular subtypes (along the x-axis) and relative distributions of genomic alterations within each molecular subtype (along the y-axis). **D.** Box plots show His as a function of genomic alterations.

**Supplementary Figure 7**. *Roudier et al.*

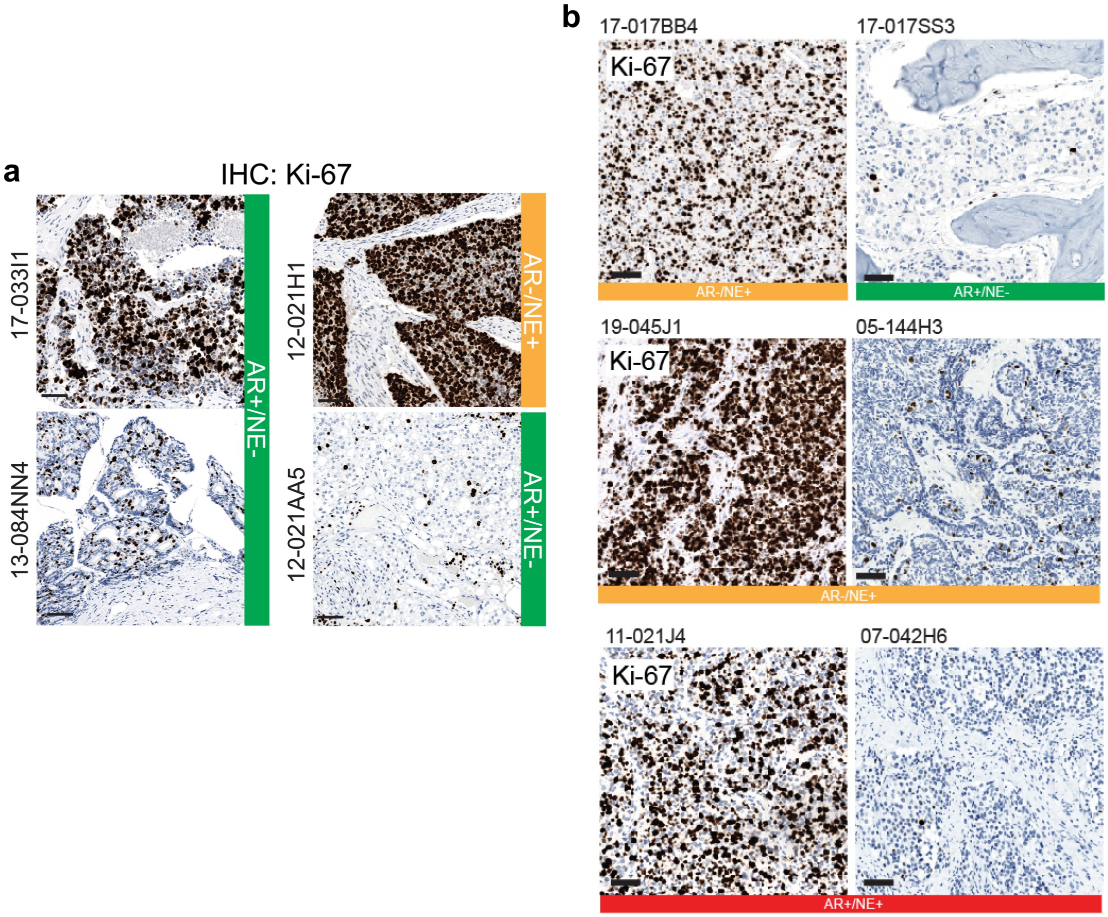


**Supplementary figure 7 A., B.** Representative micrographs show the heterogeneity of Ki-67 positivity across different patient and across individual metastases. Scale bares denote 50 $\mu$m.

**Supplementary Figure 8**. *Roudier et al.*

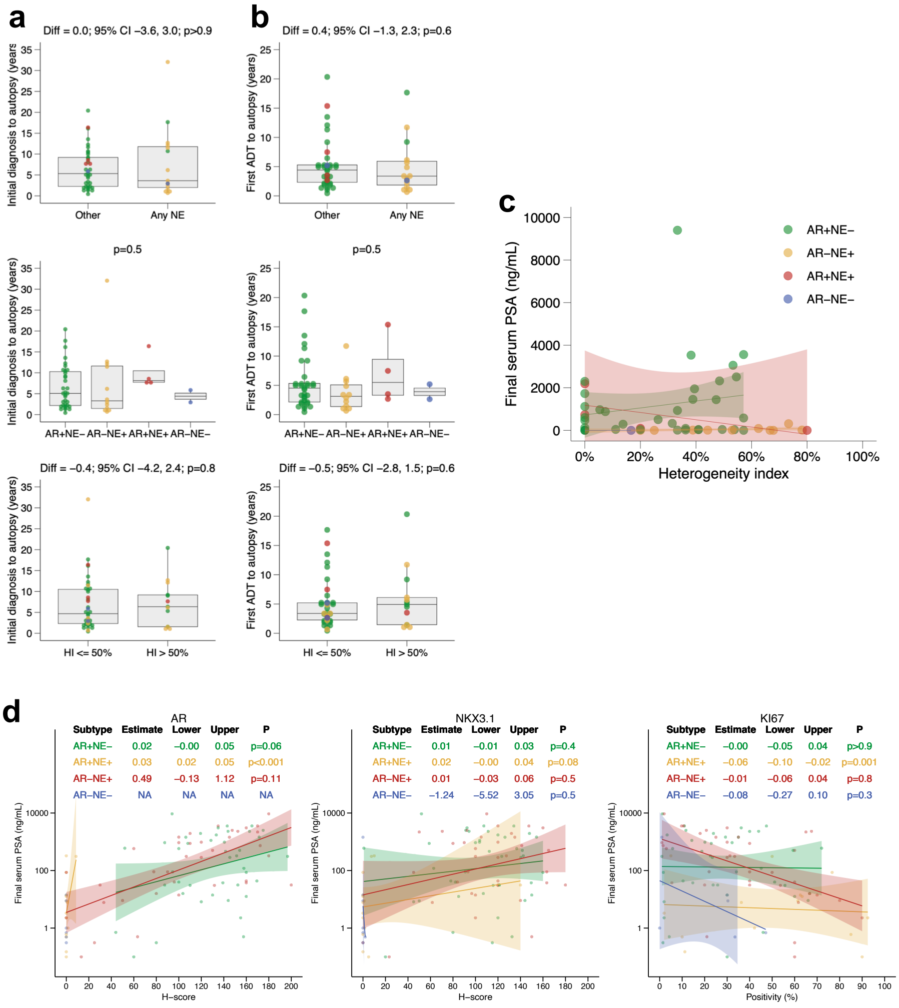


**Supplementary figure 8.** Boxplots show time intervals from initial diagnosis **A.** or first ADT **B.** to death as a function of presence or absence of NE marker expression, molecular subtype or HI > 50%. **C.** Scatter plot shows lack of correlation between heterogeneity indices and PSA levels. **D.** Scatter plots show relationships between PSA levels and AR and NKX3.1 H-scores as well as Ki-67 positivity (percent).

**Supplementary Figure 9**. *Roudier et al.*

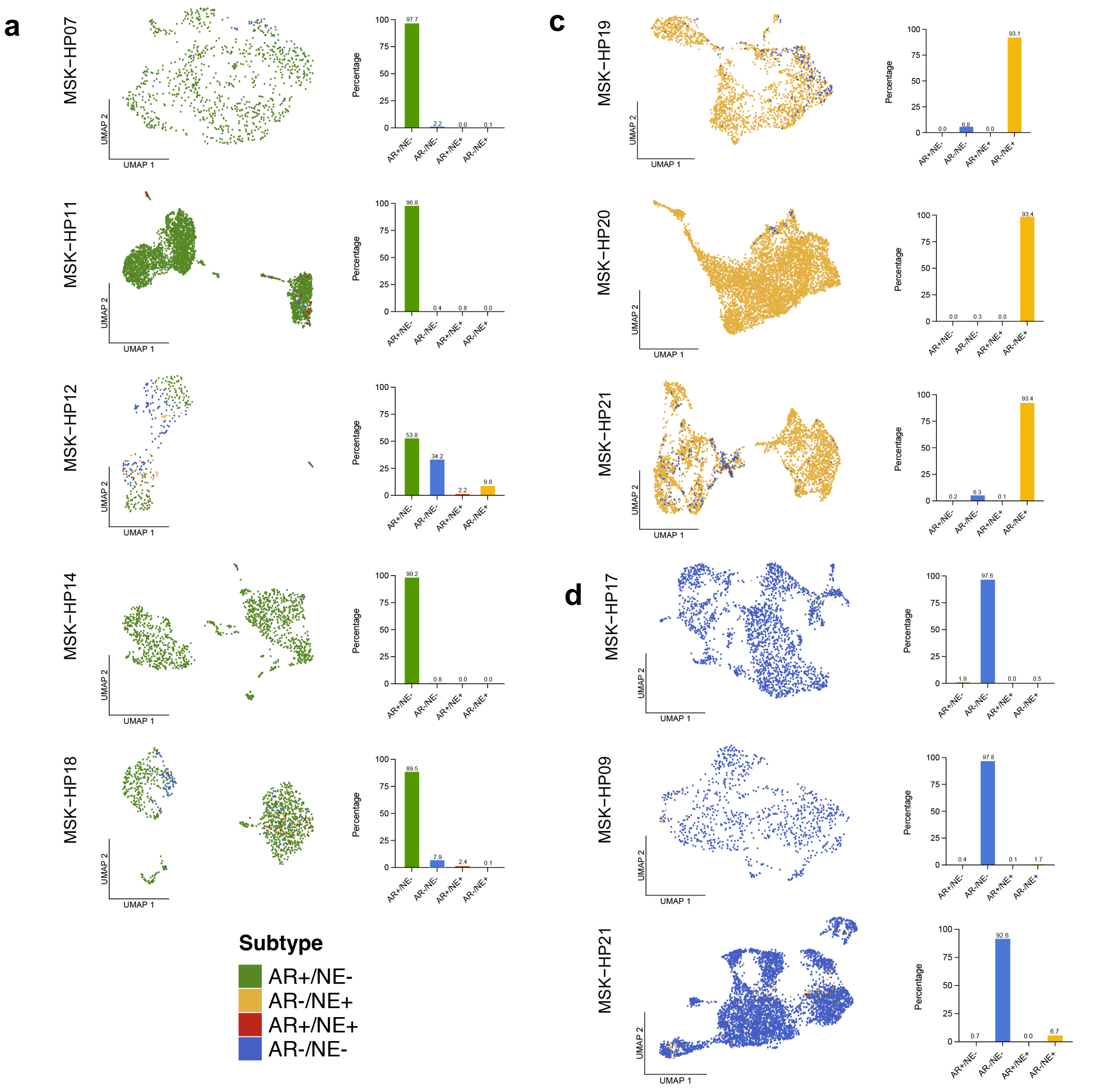


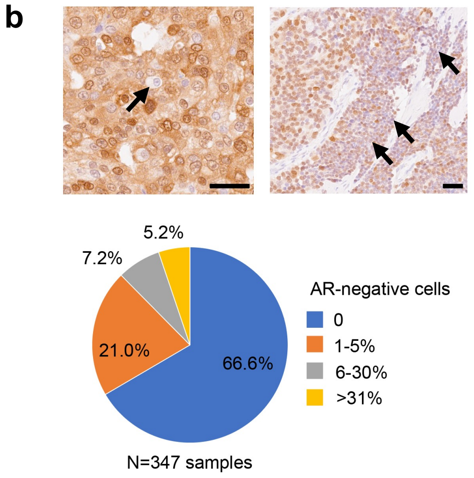


**Supplementary figure 9. A.** UMAPs depict clustered scRNA-seq data from *Zaidi et al*. for predominantly AR+/NE- cases. AR and NE positivity was assessed using established gene signatures. Bar graphs show the relative representation of each molecular subtype. **B.** IHC micrographs show staining pattern of AR+/NE- tumors. Note the AR negative cell populations (*arrows*) admixed with AR+ cells. Pie chart shows the percentage of AR negative cells across 347 AR+/NE- samples. UMAPS and bar graphs show distribution and percentages of molecular subtypes inferred from scRNA-seq data for predominantly AR-/NE+ **C.**, and AR-/NE- tumors **D.**.

**Supplementary Figure 10**. *Roudier et al.*

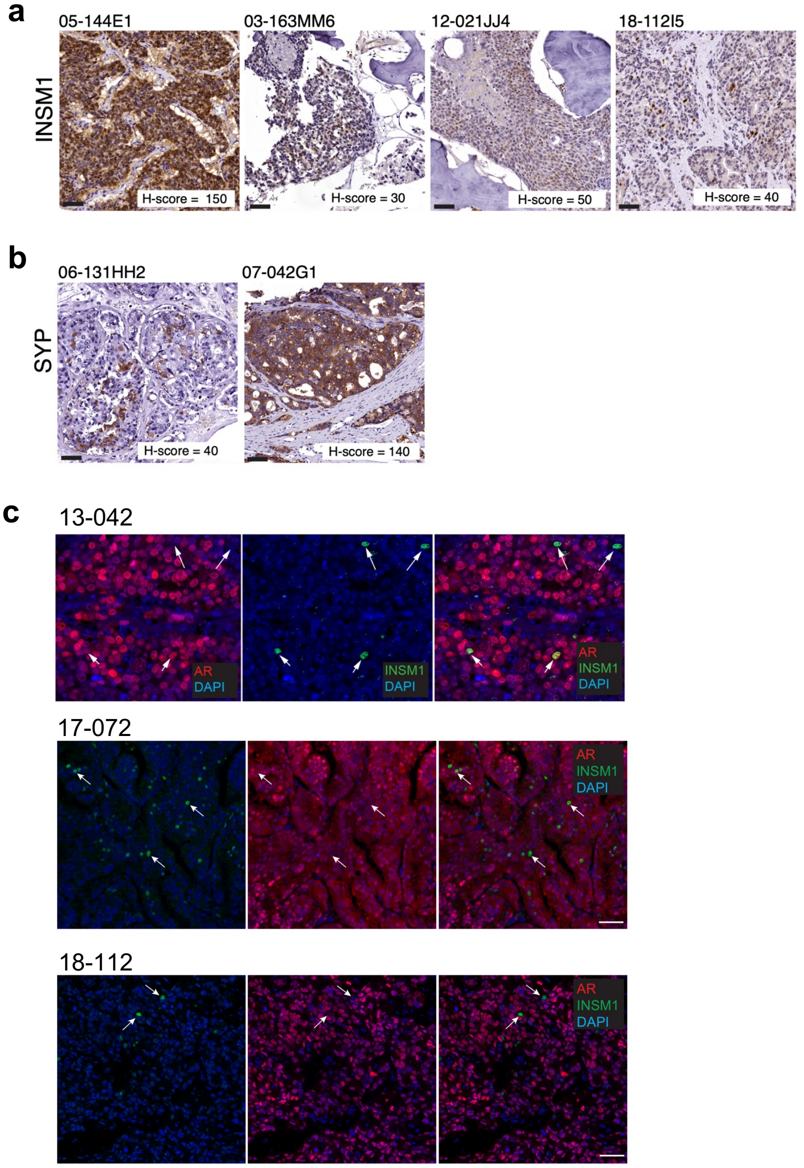


**Supplementary figure 10 A.** IHC micrographs show different patterns of INSM1 expression. **B.** IHC micrographs of SYP reactivity. **C.** Dual immunofluorescence studies show patterns of AR and INSM1 expression in mixed/bi-phenotypic tumors. Scale bars denotes 50 $\mu$m.

**Supplementary Figure 11**. *Roudier et al.*

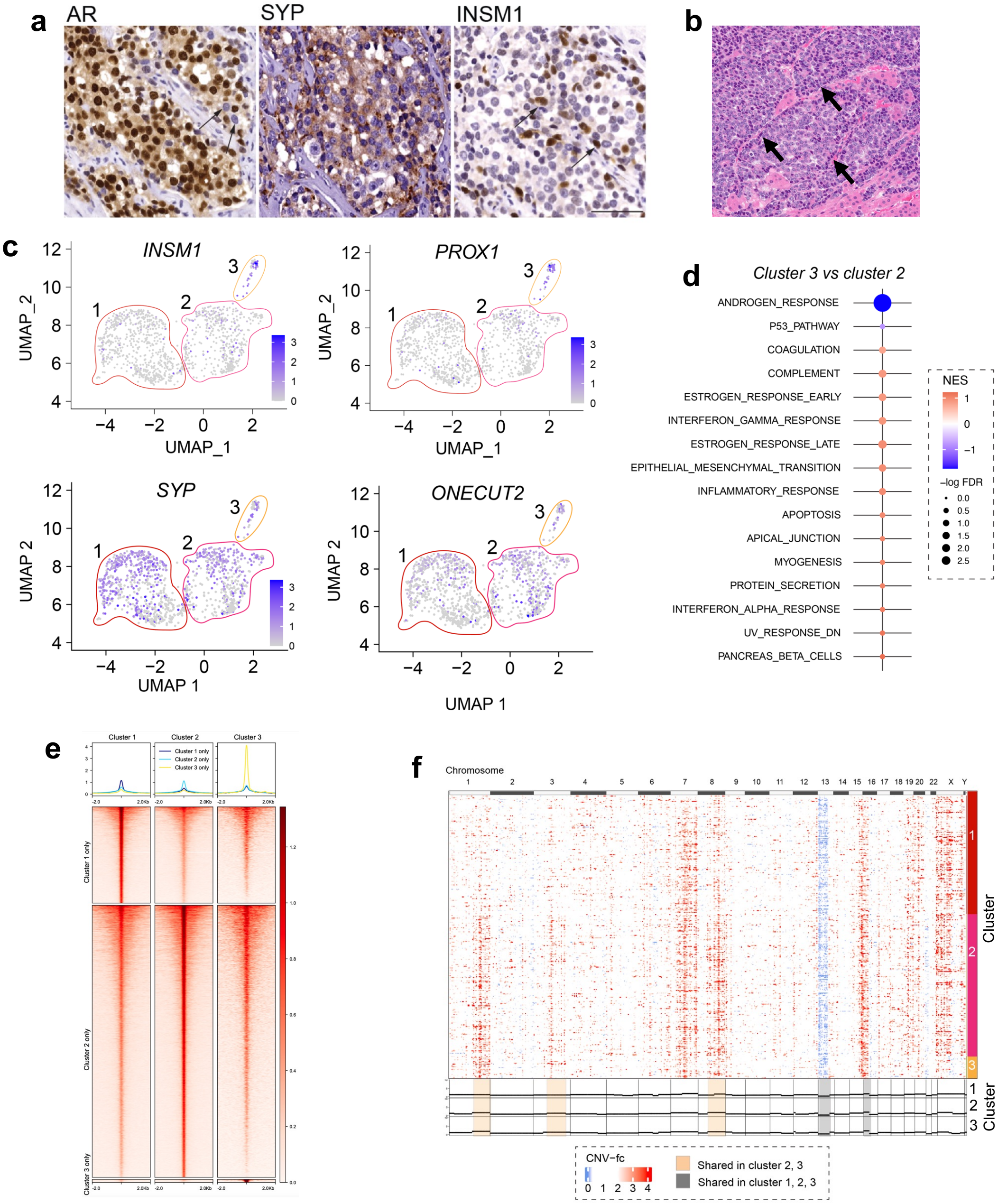


**Supplementary figure 11 A.** IHC micrographs of case 07-042 show an admixture of AR+/INSM1- and AR-/INSM1 positive cells**. B.** This biphenotypic pattern is also reflected on the morphologic level. Admixed with cells which show classic prostatic adenocarcinoma (large nuclei, open chromatin, prominent nucleoli) are cell with high nuclear to cytoplasmic ratio and dense chromatin, similar to small cell carcinoma (*arrows*). Note that these cells are located preferentially at the periphery of the tumor nests. **C.** UMAPs show the distribution of INSM1, PROX1, SYP and ONECUT2 expression across the district 3 clusters in case 07-042. **D.** Gene set enrichment analysis reveals differential expression of AR and interferon/inflammation-related Hallmark gene sets between cluster 2 vs 3. **E.** Heatmaps of centered pseudo-bulk differential chromatin accessibility sites across distinct snATAC-seq clusters. **F.** Inferred single-cell copy-number analyses, based on snATAC-seq data demonstrate the clonal relationship between all clusters and show evidence for subclonal evolution from cluster 1 to cluster 2/3. *Top*, heatmap of CNVs on the single cell level. *Bottom*, smoothed CNV tracks for all 3 clusters.

**Supplementary Figure 12**. *Roudier et al.*

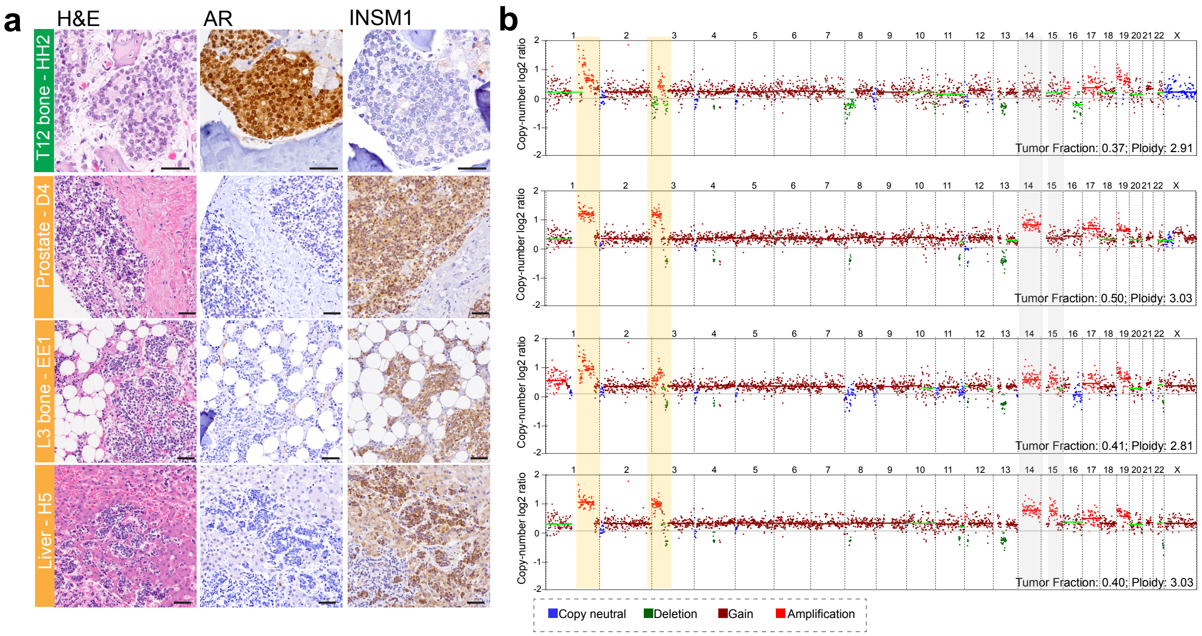


**Supplementary figure 12.** Integrative histomorphologic and genomic assessment of case 18-039. Note the extensive liver, bone and prostate involvement by AR-/NE+ high-grade neuroendocrine carcinoma. In addition, there was an isolated AR+/NE- thoracic vertebral metastasis (T12) with cribriform adenocarcinoma morphology. **A.** H&E and IHC micrographs show expression and morphologic heterogeneity across different metastatic sites. **B.** Whole genome sequencing reveals share copy-number changes (yellow) and focal subclonal copy-number gains (gray) in different metastatic sites. Scale bars denotes 50 $\mu$m.

**Supplementary Figure 13**. *Roudier et al.*

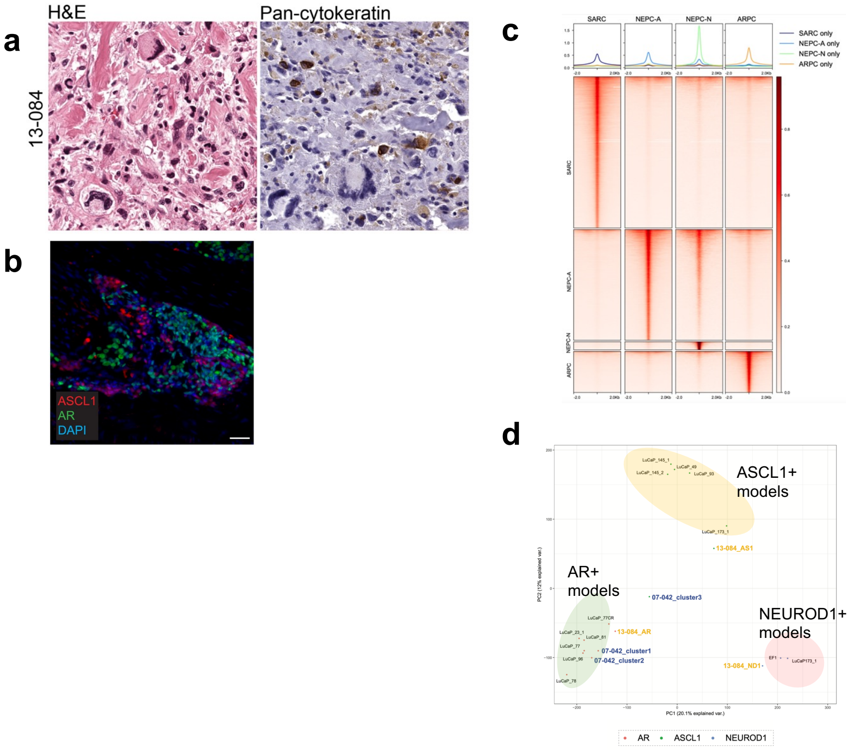


**Supplementary Figure 13. A.** H&E and pan-cytokeratin IHC micrographs of the sarcomatoid component in case 13-084. **B.** Dual immunofluorescence micrograph showing intermingling AR and ASCL1 positive cell populations. **C.** Heatmaps of centered pseudo-bulk differential chromatin accessibility sites across distinct snATAC-seq clusters. **D.** Principal component analysis shows clustering of scATAC-seq derived cell populations in the context of previously published bulk ATAC-seq profiles from phenotypically well characterized patient derived xenograft and cell line models. Note the tight clustering of the scATAC-seq samples (bold) with tumor of the respective molecular subtype. Scale bars denotes 50 $\mu$m.

**Supplementary Figure 14**. *Roudier et al.*

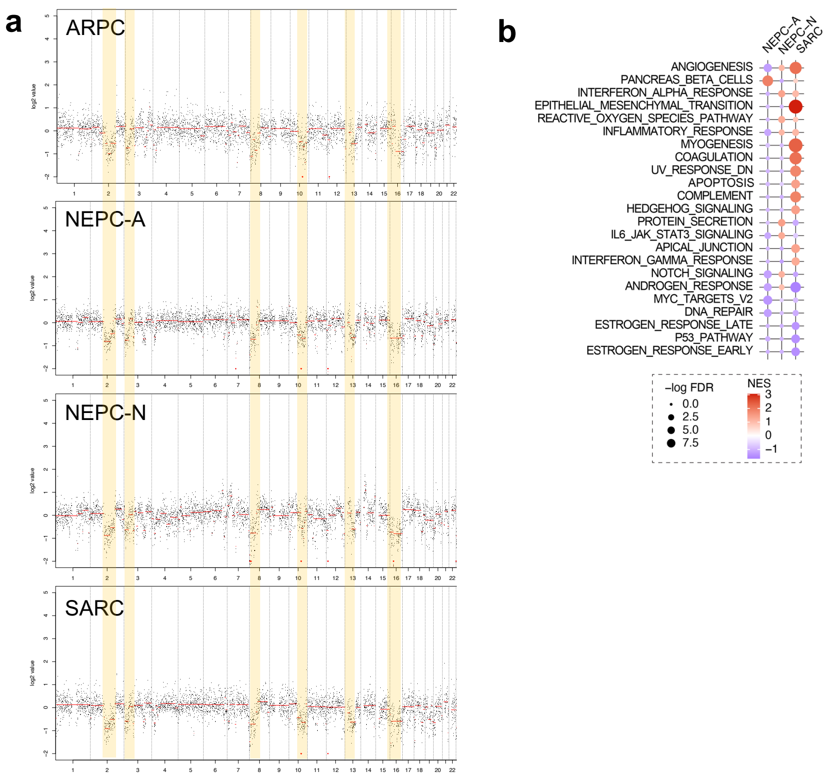


**Supplementary Figure 14. A.** Inferred single-cell copy-number analyses, based on snATAC-seq data highlight shared CNV alterations across all clusters which reflect the CNV changes found in bulk WGS data of the prostate tumor. **B.** Gene set enrichment analysis comparing the ARPC against all other clusters show differential activity of core Hallmark pathways.
